# Structures in solid state and solution of dimethoxy curcuminoids: regioselective bromination and chlorination

**DOI:** 10.1186/1752-153X-7-107

**Published:** 2013-06-25

**Authors:** Petra Galer, Amalija Golobič, Jože Koller, Berta Košmrlj, Boris Šket

**Affiliations:** 1 Faculty of Chemistry and Chemical Technology, University of Ljubljana, Aškerčeva 5, SI-1000, Ljubljana, Slovenia

**Keywords:** Dimethoxy curcuminoids, X-ray structures, NMR experiments of solids, Theoretical investigations, Regioselectivity, Enols, Halogenation, α-halodiketones

## Abstract

**Background:**

Several papers described the structure of curcumin and some other derivatives in solid and in solution. In the crystal structure of curcumin, the enol H atom is located symmetrically between both oxygen atoms of the enolone fragment with an O···O distance of 2.455 Å, which is characteristic for symmetrical H-bonds. In the solution, the geometry of the enolone fragment is attributed to the inherent disorder of the local environment, which solvates one of the basic sites better than the other, stabilizing one tautomer over the other. In this paper, how the position of methoxy groups in dimethoxy curcuminoids influence the conformation of molecules and how the halogen atoms change it when they are bonded at α-position in keto-enol part of molecules is described.

**Results:**

Six isomers of dimethoxy curcuminoids were prepared. Conformations in solid state, which were determined by X-ray single crystallography and ^1^H MAS and ^13^C CPMAS NMR measurements, depend on the position of methoxy groups in curcuminoid molecules. In solution, a fast equilibrium between both keto-enol forms exists. A theoretical calculation finding shows that the position of methoxy groups changes the energy of HOMO and LUMO. An efficient protocol for the highly regioselective bromination and chlorination leading to α-halogenated product has been developed. All α-halogenated compounds are present mainly in *cis* keto-enol form.

**Conclusions:**

The structures in solid state of dimethoxy curcuminoids depend on the position of methoxy groups. The NMR data of crystalline solid samples of 3,4-diOCH_3_ derivative, XRD measurements and X-ray structures lead us to the conclusion that polymorphism exists in solids. The same conclusion can be done for 3,5-diOCH_3_ derivative. In solution, dimethoxy curcuminoids are present in the forms that can be described as the coexistence of two equivalent tautomers being in fast equilibrium. The position of methoxy groups has a small influence on the enolic hydrogen bond. Theoretical calculations show that the energy gap between HOMO and LUMO depend on the position of methoxy groups and are lower in solution. Chlorination and bromination on α-position of 1,3-diketone moiety do not change the preferential form being cis keto-enol as in parent compounds.

## Background

The natural product curcumin (chemical name: 1,7-bis(3-methoxy-4-hydroxyphenyl)-1,6-heptadiene-3,5-dione) has been recognized for its medical properties and is utilized for the treatment of many diseases because of its anti-inflammatory, anti-oxidant, anti-viral, and anti-angiogenic properties [1-6]. Curcumin has demonstrated preventive activity against Aβ aggregation in Alzheimer’ models [7,8] and activity against various cancers [9-12] including leukaemia, liver, breast and prostate cancers.

Several papers described the structure of curcumin and some other derivatives in solid and solution. The crystal structure of curcumin was first determined in 1982 by Tønnesen et al. [13]. It was stated that the enolic H atom was statistically distributed over two oxygen atoms and each position having half occupancy. A redetermination of the crystal structure of curcumin was done by Row et al. in 2007 [14] using curcuminoid powder extracted from turmeric without further purification. They observed that the enol H atom is located symmetrically between both oxygen atoms of an enolone fragment with the O···O distance of 2.455 Å, which is characteristic for a symmetrical H-bond. It is also known from the literature that substitution of a hydroxy group with an acetoxy group, also an electron donating, in the phenyl ring of curcumin changes the crystal structure. 4-Acetoxy derivative [15] of curcumin exists in the keto-enol form in which the enol H-atom is disordered between both oxygen atoms. Authors also confirm the structure of the parent compound reported more than 20 years before by Tønnesen et al. in reference [13].

In 2011 Nangia and co-workers published the paper in which described that curcumin exists in three polymorphs and an amorphous phase. The molecule exists in the β-keto enol tautomer form in all crystal structures. In one polymorph the molecule has a slightly twisted conformation, but is linear, planar in other two polymorphs [16].

In the solution, the geometry of the enolone fragment is attributed to the inherent disorder of the local environment, which solvates one of the basic sites better than the other, stabilizing one tautomer over the other.

**Scheme 1:**
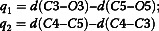
Tautomeric forms of curcumin.

Curcumin is essentially in only *cis* keto-enol form in numerous solvents, ranging from chloroform to mixtures of DMSO and water with varying pH in the range 3–9 [17-24]. Theoretical calculations have established that the *cis* keto-enol form is about 7.75 kcal mol^–1^ more stable than the *cis* diketo form [1,25]. Three possible structures of curcumin [26] can exist: the β-diketone tautomer and two equivalent asymmetric keto-enol tautomers with strong intramolecular H-bonds (Scheme 1).

The presence of halogen atoms in many natural products profoundly influences their biological activity [27]. However, in the literature only a few reports about the direct introduction of halogen atom in curcumin or curcuminoid molecules have been found [28-31]. These molecules are a relatively complex conjugate system with several active sites. Most of the β-diketones and β-keto esters that have been chlorinated, brominated and iodinated by numerous reagents and methods [32,33] usually contain one active methylene group as the only reactive site for halogenations [34-36].

We have recently developed a convenient and efficient method to introduce a halogen atom regioselectively into a methylene group in 1,3-diketone moiety or to an activated phenyl ring of 1-phenyl-3-(3,5-dimethoxyphenyl)-propane-1,3-dione using N–X reagents, such as NXS (*N*-halo succinimide), NXSacc (*N*-halo saccharin), and F-TEDA (1-chloromethyl-4-fluoro-1,4-diazoniabicyclo[2,2,2]octane bis tetrafluoroborate) or NFTh (1-hydroxy-4-fluoro-1,4-diazoniabicyclo[2,2,2]octane bis tetrafluoroborate) in the presence of LiClO_4_ and CH_3_CN as solvent [37]. We determined that introduction of a halogen atom at α-position in 1,3-dione moiety of 1-phenyl-3-(3,5-dimethoxyphenyl)propane-1,3-dione dramatically changes the conformation, being 1,3-diketone in comparison with the parent compound in which the favoured form is keto-enolic.

## Results and discussion

As it is known that electron donating groups have a quite different effect in their being bonded at orto and para or meta position in the phenyl ring we determined how the position of the methoxy groups in dimethoxy substituted curcuminoids influence the preferent tautomer form in both a solid and in a solution. We chose dimethoxy curcuminoids also for the following reasons: they contain (a) an activated methylene group in 1,3-diketone moiety, (b) a highly activated phenyl ring with two methoxy groups and (c) a double C=C bond in a conjugate position with the phenyl ring and 1,3-diketone moiety.

We also examined how the different positions of the methoxy groups is reflected in electron density distribution over the whole conjugate system and how the preferent tautomers in the solution influence the regioselectivity of halogenation. Is the activated phenyl ring or the activated methylene group the most reactive position?

### Synthesis of dimethoxy curcuminoids 2a-f

**Figure 1:**
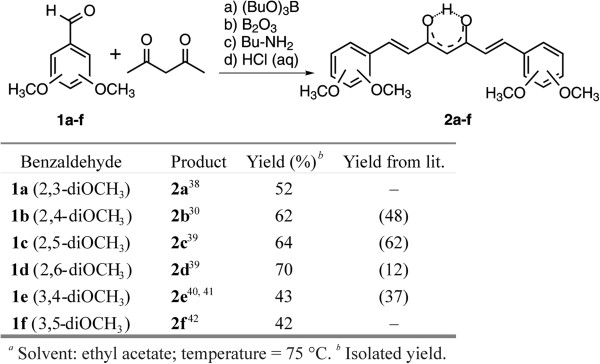
**Synthesis of compound 2**^**a**^**.**

To test the recently described protocol in the curcuminoid series, six isomers of dimethoxy derivatives were prepared using slightly modified literature procedures [30,38-42] (Figure 1). In all cases, a higher yield of curcuminoids were obtained and no additional purification by flash chromatography were necessary.

### Crystal structures of compound 2d and 2e

**Figure 2:**
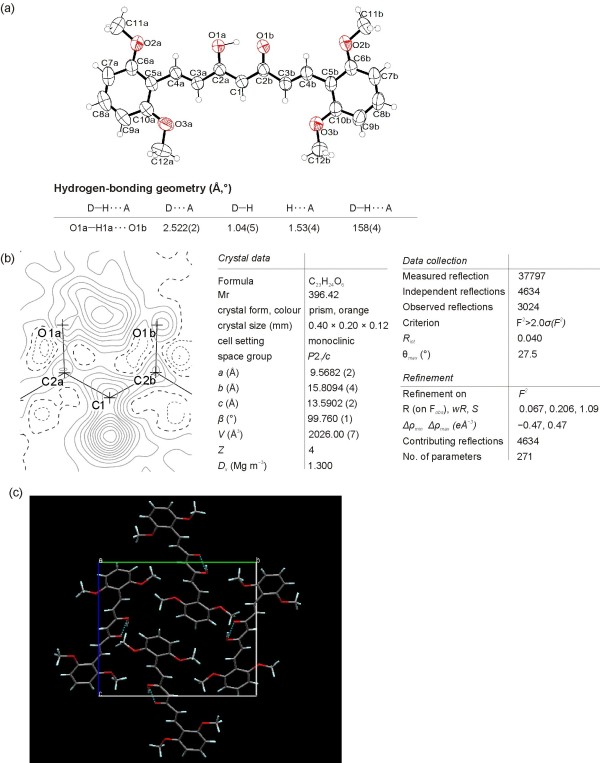
**X-ray structure of 2d. (a)** ORTEP view of 2d (50% probability displacement ellipsoids). Hydrogen atoms are drawn as circles of arbitrary radii. **(b)** Difference-Fourier map plotted in the mean plane of labelled atoms. Dotted contour lines indicate negative, dashed lines zero, and full lines positive residual electron density. **(c)** Packing of molecules of 2d as viewed along *a* axis.

The structures of dimethoxy curcuminoids in solid form were determined by X-ray crystallography. The ORTEP drawing of compound **2d** showing the asymmetric unit, which contains one 2,6-dimethoxy curcuminoid molecule (Figure 2a) in *cis* keto-enol form. A difference Fourier map (Figure 2b) shows the two largest peaks corresponding to one H atom bonded to O1a (O3)* and one H atom bonded to C1 *(C4)* (sp^2^ hybridised) in plane of enolone moiety (O1a *(O3)*, C2a *(C3)*, C1 *(C4)*, C2b *(C5)*, and O1b *(O5)*). * *In brackets and italicized, the numbering of atoms using in IUPAC nomenclature are given.* There is no peak bonded to O1b *(O5)* that would indicate a disordered H-atom of another keto-enol form. A possible explanation comes from the crystal packing in which the surrounding of O1a *(O3)* atom is different than of O1b *(O5)*, (Figure 2c). C2a–O1a *(C3–O3)* Distance (1.294(2) Å) is remarkably longer than C2b–O1b *(C5–O5)* (1.279(2) Å, ∆/σ = 7.5) and C1–C2a *(C4–C3)* (1.380(3) Å) is quite shorter than C1–C2b *(C4–C5)* (1.405(3) Å, ∆/σ = 13). In contrast, both lengths C2a–O1a *(C3–O3*) and C1–C2b *(C4–C5)* are remarkably shorter than the corresponding pure single bonds (C–O ≈ 1.37 and C–C ≈ 1.48 Å) and the pairs of bonds C2b–O1b *(C5–O5)* and C1–C2a *(C4–C3)* are longer than the corresponding typical double bonds (C=O ≈ 1.20 and C=C ≈ 1.33 Å). The donor of the intramolecular hydrogen bond is O1a *(O3)* and the acceptor O1b *(O5*). The inter-atomic distances O1a···O1b *(O3*···*O5)*, O1a–H1a *(O3–H3)* and O1b···H1a *(O5*···*H3)* are 2.522(2) Å, 1.04(5) Å and 1.53(4) Å, respectively. The O1a–H1a···O1b *(O3–H3*···*O5)* angle is 158(4)°.

*π*-Delocalization into enolone fragment was determined from *π*-delocalization index λ_*Q*_ calculated with Equations 1, 2, 3 and 4, using the experimental determined bond distances [43]. λ_*Q*_ = 0.437, showing that the degree of delocalization is approximately 87%.

(1) $$q_{1}=d\left(C3–O3\right)–d\left(C5–O5\right);q_{2}=d\left(C4–C5\right)–d\left(C4–C3\right)$$

(2) $$Q=q_{1}+q_{2}$$

(3) $$\lambda =\lambda_{Q}=\left(1–Q/Q_{o}\right)/2\;\mathrm{and}\;Q_{o}=0.320\;Å$$

(4) $$\begin{array}{l} \mathrm{Percentage}\;\mathrm{of}\;\mathrm{delocalization}:\\ \mathrm{Del}\%=100\;\left(1–|2\;\lambda –1|\right) \end{array}$$

The molecules deviate from planarity: the planes through dimethoxy aromatic rings are for 6.59(5) and 9.48(5)° out of plane through the central, enolic part of molecules.

**Figure 3:**
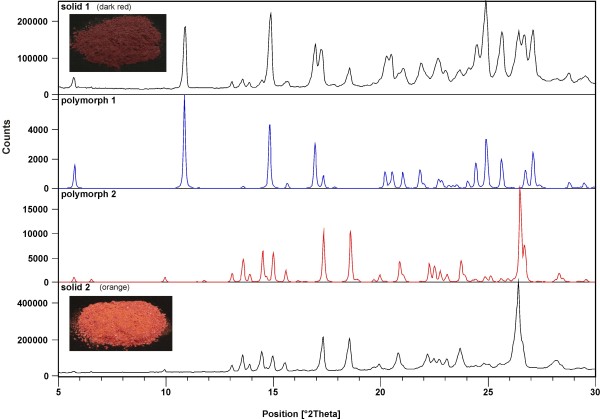
**XRD diagrams of 2e.** Measured pattern of solid 1, solid 2 and calculated pattern from X-ray structure of polymorph 1, polymorph 2.

The crude product of **2e** was further purified in two different ways. (a) To the crude product, EtOAc was added, insoluble material was filtered off, and the solvent was evaporated under reduced pressure. The oily product obtained was slowly crystallized. Under vigorous stirring methanol was added to eliminate the impurities. Dark red color crystals, from suspension, were filtered off (termed solid 1). (b) To the crude product water was added to eliminate the inorganic materials. After extraction with EtOAc and evaporation of the solvent under reduced pressure an orange colored solid was obtained (termed solid 2) (Figure 3). From both solids single-crystals (polymorph 1 and 2) were obtained and X-ray structures were done.

**Figure 4:**
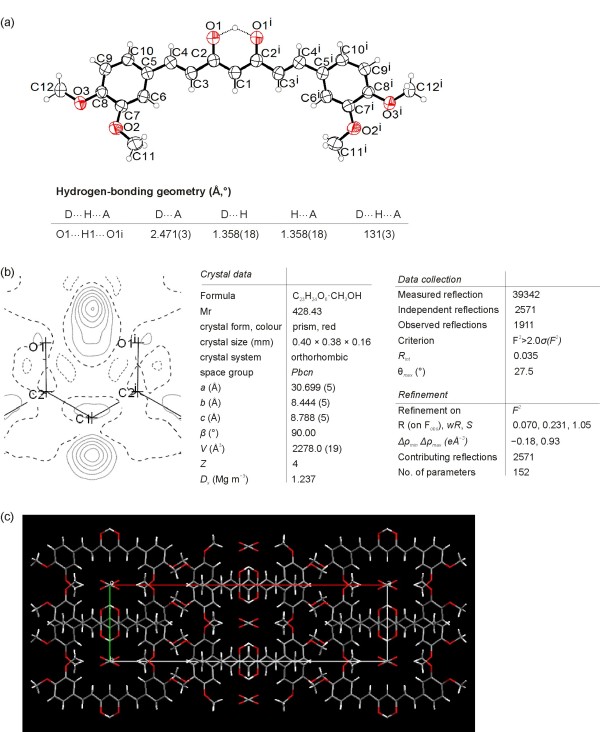
**X-ray structure of 2e, polymorph 1. (a)** ORTEP view of 2e, polymorph 1 (50% probability displacement ellipsoids). Hydrogen atoms are drawn as circles of arbitrary radii. [Symmetry code (i) –x, y, –z+12] **(b)** Difference-Fourier map plotted in the mean plane of labelled atoms. **(c)** Packing of molecules of 2e (polymorph 1) as viewed along *c* axis. In the channels oriented along *c* axis are also molecules of solvent, methanol.

The crystal structure of polymorph 1 consists of the symmetric 3,4-dimethoxy curcuminoid molecules (Figure 4a). One half of the molecule is related symmetrically to the other half by the twofold axis. A difference Fourier map of polymorph 1 (Figure 4b) clearly shows the two peaks in the plane of O1 *(O3)*, C2 *(C3)*, C1 *(C4)*, C2^i^*(C5)* and O1^i^*(O5)* located on a twofold axis (i: –x, y, –z+12): one corresponding to H atom bonded to C1 *(C4)* (sp^2^ hybridised), excluding the diketo form, and another one representing enol hydrogen atom centred between O1 *(O3)* and O1^i^*(O5)* at the distance 1.358(18) Å from both of O atoms. Contrary to structure of **2d** both O atoms (O1 *(O3)* and O1^i^*(O5)*) are due to the symmetry in the same crystal surrounding, see Figure 4c. Polymorph 1 forms a very short O1···O1i *(O3*···*O5)* contact distance, 2.471(3) Å. Appropriate pairs of bonds C1–C2 *(C4–C3)*, C1–C2i *(C4–C5)* and C2–O1 *(C3–O3)*, C2i–O1i *(C5–O5)* are identical in length, 1.397(3) and 1.290(3) Å, respectively. The O1···H1···O1i *(O3*···*H3*···*O5)* angle is 131(3)°. Such a symmetrical and delocalized structure of molecules was also obtained by checking with structure determination in space groups *Pbc*2_1_ and *P*2_1_*cn*, when the whole molecule was taken as the asymmetric unit (Additional file 1: Figure S4).

Calculated parameter *Q* = 0; λ_*Q*_ = 0.500 (of polymorph 1) corresponding to the totally π-delocalized enolone fragment that is indicative of a symmetric single-well H-bond, typical of strong bond [44,45]. The molecules deviate from planarity: the dihedral angle between the plane through the dimethoxy aromatic ring and the plane through the central, enolic part of molecule is 18.33(9)°.

**Figure 5:**
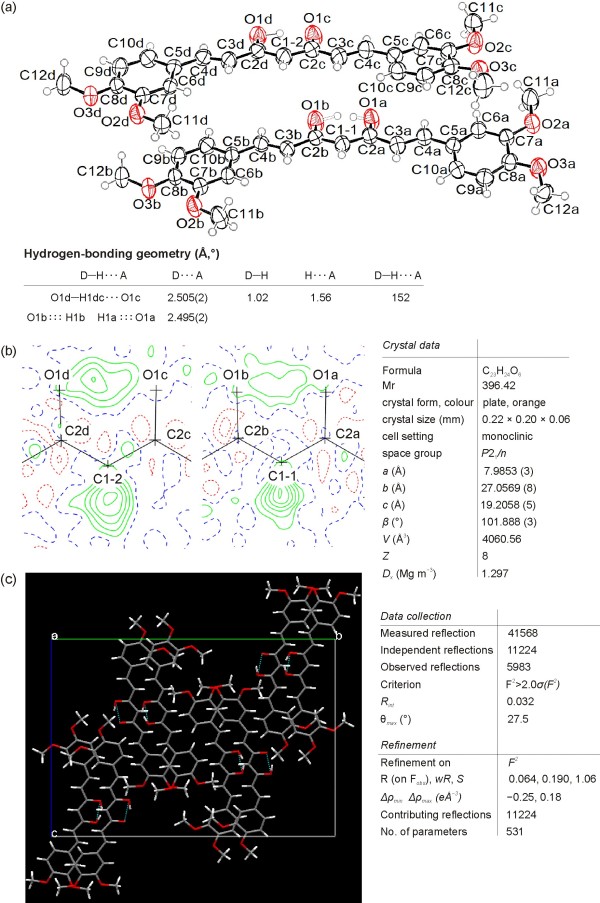
**X-ray structure of 2e, polymorph 2. (a)** ORTEP view of 2e, polymorph 2 (50% probability displacement ellipsoids). Hydrogen atoms are drawn as circles of arbitrary radii. **(b)** Difference-Fourier maps plotted in the mean plane of labelled atoms. **(c)** Packing of molecules of 2e (polymorph 2) as viewed along *a* axis.

The ORTEP drawing of polymorph 2 showing the asymmetric unit, which contains two molecules, is presented in Figure 5a. A difference Fourier map (Figure 5b) of first molecule shows that an enolic H atom is bonded to O1d *(O3)*, there is no peak bonded to O1c *(O5)*. Distance C2d–O1d *(C3*–*O3)* 1.297(3) Å is longer than C2c–O1c *(C5*–*O5)* 1.288(3) Å and C1-2–C2d *(C4*–*C3)* 1.384(3) Å is shorter than C1-2–C2c *(C4*–*C5)* 1.396(3) Å. Next, a difference Fourier map of second molecule from the asymmetric unit shows two maxima, approximately 1Å from O1b *(O3)* and O1a *(O5)* corresponding to two enolic H atoms with 50% occupancy. Contrary to the first molecule in the asymmetric unit, the distances C2b–O1b *(C3*–*O3)* 1.286(3) Å and C2a–O1a *(C5*–*O5)* 1.287(3) Å on one side and C1-1–C2b *(C4*–*C3)* 1.394(3) Å and C1-1–C2a *(C4*–*C5)* 1.392(3) Å on the other side are nearly the same. A comparison between the above bond lengths in enolone moiety shows that the π-delocalization must be higher in second molecule than in the first. π-Delocalization into enolone fragment was determined for both molecules in single crystal of polymorph 2. λ_*Q*_ Index calculated from experimental determined bond distances is 0.467 for first molecule, corresponding to 93% of delocalization and 0.495 for second molecule showing that the degree of delocalization is 99%.

Parameters of hydrogen-bonding geometry are collected in the table Figure 5. It is also important to emphasize the difference between polymorph 1 and polymorph 2 in orientation one of the substituted phenyl rings (see Figures 4 and 5). Comparing the experimental powder XRD pattern for solid 1 and 2 with those of calculated lines from the crystal structure of polymorph 1 and 2 clearly shows that solid 1 contains both polymorphs while solid 2 is pure polymorph 2 (Figure 3). To our surprise, experimental XRD pattern of solid 3, standing at room temperature for a long period of time, shows lines corresponding neither to polymorph 1 nor to polymorph 2 (Additional file 1: Figure S2).

### Solid-state NMR measurements of 2d, 2e and 2f

**Figure 6:**
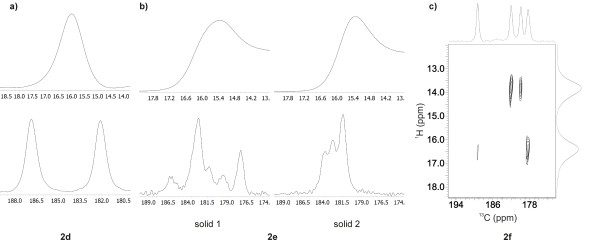
**NMR spectra of solid samples of 2d, 2e and 2f. (a)**, **(b)**^1^H echo MAS NMR spectra (up) and ^1^H-^13^C CPMAS NMR spectra (down) for solid sample of 2d and 2e (solid 1, solid 2), respectively. **(c)** 2D ^1^H-^13^C HETCOR NMR spectrum of compound 2f. NMR spectra of solid samples present only enolone region and were recorded on 600 MHz NMR spectrometer.

It is necessary to state that the X-ray structure shows the picture of a single crystal and that the situation in the solid sample can be different if polymorphism exists. For this reason, ^1^H MAS and ^13^C CPMAS NMR experiments were carried out. In ^1^H MAS NMR spectrum of 2,6-diOCH_3_ derivative (**2d**), one signal for enolic proton appears at 15.99 ppm and in ^13^C CPMAS NMR spectrum; in addition to other signals, two signals at 186.9 and 182.1 ppm corresponding to two different carbon atoms of the enolic part of molecule (Figure 6a). As the chemical shifts are very close to each other, the asymmetry of H-bond in enolone moiety is not strongly expressed. The data obtained are in accordance with X-ray structure.

In the ^13^C CPMAS NMR spectrum of 3,4-diOCH_3_ (**2e**) (Figure 6b), the sample of crystalline solid 2 (orange, Figure 3), a structured overlapped signal appears between 180.0 and 185.0 ppm, corresponding to carbonyl carbon atoms of enolone moiety showing similar character and the fact is that an asymmetry in those parts of molecule are not strongly expressed. A relatively high degree of delocalization of both molecules confirm this conclusion. The data obtained are in accordance with the X-ray structure of polymorph 2 and the XRD pattern showing the presence of only one phase.

Solid 1 (dark red, Figure 3) contains two polymorphs, form 1 and form 2, and a small amount of an amorphous phase as determined from the XRD measurement. ^13^C CPMAS NMR spectrum is rather complicated, with several signals in the region characteristic for carbonyl carbon atoms. By comparing ^13^C CPMAS NMR spectra of solid 1 and solid 2 we are able to establish which signals probably belong to polymorph form 1. At the beginning we were surprised to find such a complex spectrum, as we expected due to the symmetry in molecules of polymorph 1 only one signal for carbonyl carbon atoms of enolone moiety. From the X-ray analyses we have learned that the polymorph 1 is solvate, contains molecules of methanol which are disordered along the channels and therefore can randomly interact with the enolone part of the molecule and lead to different chemical shifts for carbonyl carbon atoms.

^1^H MAS and ^13^C CPMAS NMR spectra of 3,5-diOCH_3_ (**2f**) (Figure 6c) indicate that two different tautomers are present in the solid sample. On the basis of ^1^H-^13^C 2D HETCOR spectrum we determined that one tautomer with an enolic proton at 16.39 ppm correlates to ^13^C CPMAS NMR spectrum with two signals at *δ* = 178.8 and 189.8 ppm belonging to the carbon atom of the enolone part of the molecule. Relatively great differences in chemical shifts indicates a different character of both carbonyl atoms. A signal at higher chemical shifts (189.8 ppm) belongs to an atom of the carbonyl group while the signal at a lower chemical shift (178.8 ppm) belongs to an enolic carbon atom.

In the second tautomer (in ^1^H MAS NMR spectrum two signals for enolic protons are present) signals at 182.5 and 180.4 ppm correlate with the enolic proton signal at 13.85 ppm. A smaller difference between signals for both carbon atoms of the enolone part of the molecule reflects its similar character probably due to the higher symmetry in the enolone part of molecule.

### Structures of compounds 2 in solution

**Figure 7:**
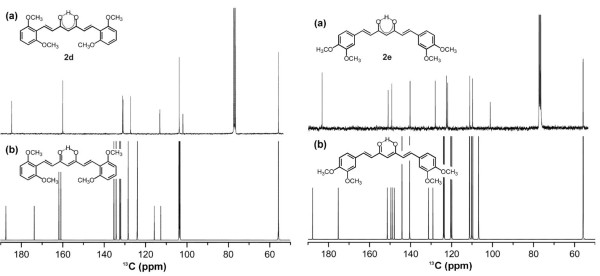
**Measured and computed**^**13**^**C NMR spectra for 2d and 2e. (a)**^13^C NMR spectrum in CDCl_3_ solution and **(b)** prediction of *cis* keto-enol form of compound 2d (left) and 2e (right).

The preferred structures of dimethoxy curcuminoids in solution were examined by ^1^H, ^13^C NMR (Additional file 2) and UV–VIS spectroscopy. In the CDCl_3_ solution, only the presence of the *cis* keto-enol form of **2** has been found. The symmetric conformations of enolone fragment of molecules were determined on the basis of equal chemical shifts for both carbonyl carbon atoms in the ^13^C NMR spectra. The ^13^C NMR prediction (^13^C NMR were predicted by ACD/NMR Processor and NMR Workbook Suite v.12. program) of the *cis* keto-enol form of 2,6-dimethoxy curcuminoid **2d** shows for enolic moiety two signals at 186.6 and 173.1 ppm and for **2e** derivative at 187.4 and 175.0 ppm (Figure 7b). In the obtained ^13^C NMR spectra, separate signals are not seen because of rapid tautomerization between both tautomers. Instead, only one signal is seen at 184.7 ppm for **2d** and at 183.2 ppm for **2e** (Figure 7a). The fact that both polymorph forms of **2e** gave the same ^13^C NMR spectra in solution once again confirms the thesis that the situation in solution is quite different from those in solid state.

As the chemical shifts for carbonyl atom in other isomers are also very similar to those of **2d** and **2e**, see Table 1, the substituents bonded at the carbonyl atom, being in our cases different only in position of methoxy groups in the phenyl ring, have only a small influence on the conformation of the central enolone fragment in the solution. The small influence of substituents on the conformation of the enolone fragment can also be seen from relative small difference for chemical shifts of the enolic proton, being in all cases between 15.83 and 16.23 ppm.

**Table 1:** **Chemical shifts for enolic H-atom and carbonyl carbon atom in*cis*keto-enol form of compounds 2, 3 and 4 in CDCl**_**3**_**solution**

**Comp.**	***δ*(OH)**	***δ*(CO)**	**Comp.**	***δ*(OH)**	***δ*(CO)**	**Comp.**	***δ*(OH)**	***δ*(CO)**
**2a**	15.91	183.6	**3a**	16.41	180.5	**4a**	16.86	181.3
**2b**	16.19	183.8	**3b**	16.78	180.6	**4b**	17.21	181.5
**2c**	15.96	183.6	**3c**	16.52	180.5	**4c**	16.96	181.4
**2d**	16.23	184.7	**3d**	16.77	181.6	**4d**	17.19	182.4
**2e**	16.02	183.2	**3e**	16.62	180.1	**4e**	17.05	181.0
**2f**	15.83	183.2	**3f**	16.34	180.1	**4f**	16.74	181.0

**Figure 8:**
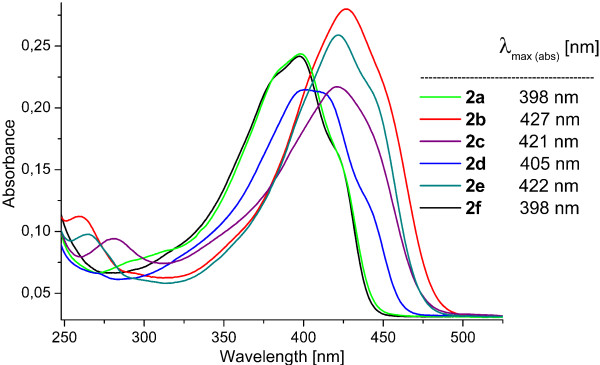
**UV–VIS spectra of compounds 2 in CH**_**2**_**Cl**_**2 **_**(5.0·10**^**–6 **^**mol/L).**

Using the correlation of chemical shift for the enolic proton and hydrogen-bond strength, we can establish that the positions of the methoxy groups have little influence on the enolic H-bond strength. To establish how the position of methoxy groups bonded in a phenyl ring influence the conformation of whole molecules, UV–VIS spectra of all dimethoxy curcuminoid isomers were carried out using 5.0·10^–6^ M CH_2_Cl_2_ solution (Figure 8). Isomers 2,3-diOCH_3_ (**2a**); 2,6-diOCH_3_ (**2d**) and 3,5-diOCH_3_ (**2f**) have absorption maximums at shorter wavelengths (but very close to each other), while the absorption maximum for 2,4-diOCH_3_ (**2b**); 2,5-diOCH_3_ (**2c**) and 3,4-diOCH_3_ (**2e**) are at longer wavelengths. Let us suppose that the influence of the methoxy groups bonded in a phenyl ring (strong electron donating group) at orto and para positions are higher from that of a meta position. For this reason, it is very difficult to maintain that only the positions of 2, 4 and 6 in dimethoxy curcuminoids contribute more to the π-delocalization of whole molecule than positions 3 and 5. The planarity of molecules is also important and must be taken into account for the explanation the differences of absorption maximum in UV–VIS spectra. When the absorption spectra were carried out in 5.0·10^–6^ M CH_3_CN solution, a small hypsochromic effect was observed (cca. 2–4 nm).

*Cis* keto-enol tautomers of dimethoxy curcuminoids in solution can be characterized by resonance-assisted intramolecular hydrogen bond and a low barrier potential well in the proton transfer pathway between two equivalent tautomeric forms. The H-centred conformation represents the transition state. Identical C–O groups of the enolone fragment guarantee the equal basicity of both oxygen atoms, necessary for equal sharing of the proton.

### Theoretical investigation of compounds 2a-f

**Figure 9:**
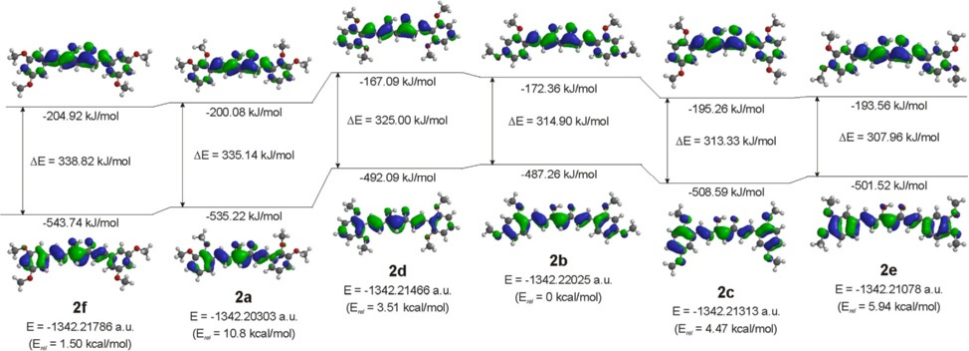
Molecular orbital diagram levels for HOMOs and LUMOs in order of decreasing HOMO-LUMO gaps, computed total energies, and relative energies of compounds 2.

To clarify the reason for changes in absorption maximum in the UV–VIS spectra when the position of methoxy groups are different in curcuminoids, the theoretical calculation in the gas phase with optimized geometries in the 6-31G(d,p) basis at the DFT/B3LYP level of theory were carried out. We determined that the total energy is different and is the highest in the case of the 2,3-diOCH_3_ (**2a**) isomer and the lowest in the case of the 2,4-diOCH_3_ (**2b**) isomer. The relative energies for all isomers in comparison to 2,4-diOCH_3_ (**2b**) are also given, see Figure 9.

On the basis of the HOMO and LUMO analyses, it can be seen that the HOMO is delocalized over the entire structure in a π molecular orbital while LUMO, which is predominantly π* in character, also shows considerable delocalization (Figure 9). The oxygen of both methoxy groups are involved in the HOMO except in the case of 2,3-diOCH_3_ (**2a**) in which only the oxygen atoms on position 2 is slightly involved and in 3,5-diOCH_3_ (**2f**) in which HOMO practically does not involve both of methoxy groups. In contrast, in LUMO the oxygen of methoxy group bonded at position 4 in the phenyl ring slightly participates in the case of 2,4-diOCH_3_ (**2b**) and 3,4-diOCH_3_ (**2e**) isomers. Small participation of electrons from the oxygen of the methoxy groups can also be seen from calculated natural atomic charges being in all cases very similar (−0.52 ± 0.01), except in the case of 2,3-diOCH_3_ (**2a**) in which the charge an oxygen atom of the methoxy group bonded at position 2 and 3 is slightly higher, –0.55.

The energy gaps between HOMO and LUMO are similar for 2,3-diOCH_3_ (**2a**), 2,6-diOCH_3_ (**2d**), and 3,5-diOCH_3_ (**2f**) on one side and for 2,4-diOCH_3_ (**2b**), 2,5-diOCH_3_ (**2c**), and 3,4-diOCH_3_ (**2e**) on the other side, and decrease from 3,5-diOCH_3_ (**2f**) (338.82 kJ/mol) to 3,4-diOCH_3_ (**2e**) (307.96 kJ/mol). These calculated data are in nearly agreement with experimental observation in UV–VIS spectra, in which the absorption maximums for 2,3-diOCH_3_ (**2a**), 2,6-diOCH_3_ (**2d**) and 3,5-diOCH_3_ (**2f**) appear at lower wavelength than the absorption maximums for 2,4- (**2b**); 2,5- (**2c**) and 3,4-diOCH_3_ (**2e**) isomers, see Figure 8.

**Figure 10:**
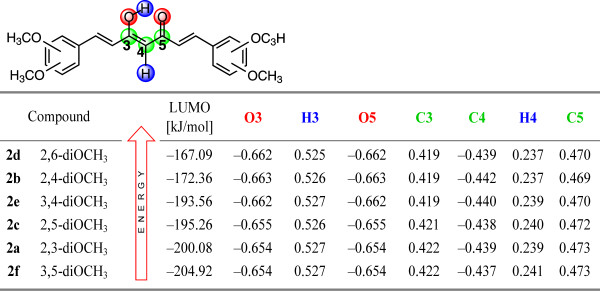
Computed LUMO energies and natural atomic charges for the enolone fragment of 2.

Natural atomic charges computed for the enol isomers of curcuminoids in the gas phase at both oxygen atoms of the carbonyl group correlate with the energy of LUMO. The charges are higher in the case of the 2,6-diOCH_3_ (**2d**) isomer with the highest LUMO energy (−167.09 kJ/mol) and lower for the 3,5-diOCH_3_ (**2f**) isomer in which the LUMO energy is the lowest and the stabilization the most pronounced. All natural atomic charges for the enolone part of dimethoxy curcuminoid isomers are given in the Figure 10.

Comparison computed HOMO and LUMO orbitals of syn-enol form of curcumin in the gas phase [17] with HOMO, LUMO orbitals of dimethoxy curcuminoids shows, firstly, that in both cases the HOMO is delocalized over the entire structure and, secondly, that the methoxy groups in curcumin are slightly involved in the HOMO, while the contribution of methoxy groups in dimethoxy curcuminoids depend on its position. In all mentioned cases, the methoxy groups practically do not participate in LUMO, as expected. The HOMO-LUMO gap of curcumin (313.6 kJ/mol) is nearly the same as in 2,5-diOCH_3_ (313.3 kJ/mol) and 2,4-diOCH_3_ (314.9 kJ/mol), slightly higher than in 3,4-diOCH_3_ (308.0 kJ/mol) and lower than in other isomers.

Energies obtained at the optimized geometries in the aqueous solution and the gas phase using DFT/B3LYP and 6311-G(2d,2p) basis sets for syn-enol tautomer of curcumin show that the total energy stabilization in water is 19.5 kcal/mol (81.9 kJ/mol), the dipole moment change from 7.67 D to 10.77 D, while the HOMO-LUMO gap is lower in the water solution by 0.18 eV (from 3.25 to 3.07 eV) [17].

From that reason we found it necessary to compute total energies, dipole moments and HOMO-LUMO gaps for all isomers of dimethoxy curcuminoids in CH_2_Cl_2_ solutions.

As seen from Table 2 the total energy stabilization of syn-enolic form of all dimethoxy curcuminoids in CH_2_Cl_2_ are only slightly different and varied from 63.1 to 70.8 kJ/mol and is lower than in the case of curcumin in water [17] which can be attributed to a lower dipole moment of dimethoxy curcuminoids in dichloromethane besides the smaller polarity of dichloromethane.

**Table 2:** **Computed total energies, total energy stabilization, homo-lumo gaps and dipole moments of dimethoxy curcuminoids 2 in gas phase and CH**_**2**_**Cl**_**2**_**solution**

**Comp.**		**Energy [kJ/mol]**	**Relative energy [kJ/mol]**	**HOMO [kJ/mol]**	**LUMO [kJ/mol]**	**HOMO-LUMO [kJ/mol]**	**λ**_***max***_**[nm]**	**μ [D]**
**2a**	vacuum	−3523956.5	0.0	−535.2	−200.1	335.1	356.94	4.47
	CH_2_Cl_2_	−3524019.7	−63.1	−548.8	−221.0	327.8	364.97	5.12
**2b**	vacuum	−3524001.7	0.0	−487.3	−172.4	314.9	379.89	0.55
	CH_2_Cl_2_	−3524072.5	−70.8	−498.8	−199.6	299.2	399.81	1.02
**2c**	vacuum	−3523983.0	0.0	−508.6	−195.3	313.3	381.79	2.15
	CH_2_Cl_2_	−3524048.2	−65.2	−512.9	−217.8	295.1	405.46	2.72
**2d**	vacuum	−3523987.1	0.0	−492.1	−167.1	325.0	368.08	4.01
	CH_2_Cl_2_	−3524055.6	−68.5	−511.1	−195.2	315.9	378.65	5.03
**2e**	vacuum	−3523976.9	0.0	−501.5	−193.6	307.9	388.45	3.57
	CH_2_Cl_2_	−3524044.3	−67.4	−505.4	−215.2	290.2	412.22	4.49
**2f**	vacuum	−3523995.5	0.0	−543.7	−204.9	338.8	353.07	4.99
	CH_2_Cl_2_	−3524061.7	−66.2	−551.4	−225.5	325.9	367.03	6.17

For all isomers of dimethoxy curcuminoids dipole moments are higher in the solution than in the gas phase. As the computationally determined HOMO-LUMO gaps in CH_2_Cl_2_ solution in comparison with those in the gas phase are lower for all isomers the absorption maxima are red shifted. But it is necessary to mention that much better correlations are obtained by direct comparison of the observed and calculated (using TDDFT/B3LYP and 631-G(d,p) basis sets) absorption maxima in UV–VIS spectra (Additional file 1: Figure S1).

### Halogenation of compounds 2a-f

**Figure 11:**
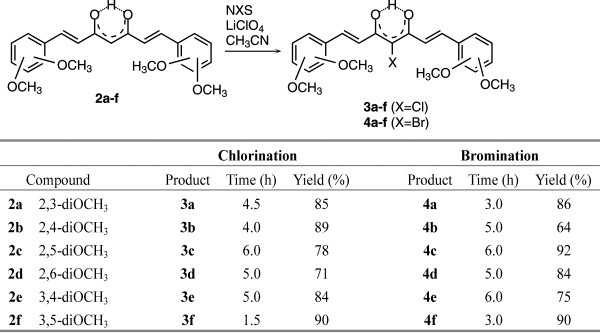
Halogenation of compound 2.

Why halogenations of dimethoxy curcuminoids? There are several reasons. a) These compounds have two reactive positions for electrophilic halogenations: the activated phenyl ring and the activated methylene group. b) If halogenation takes place on the activated phenyl ring we will be able to determine how the halogen atom participates in electron density distribution in a conjugated system of curcuminoids. c) If halogenation takes place on an activated methylene position we will be able to determine how the halogen atom influences the preferent tautomers of dimethoxy curcuminoids in solid phase and in solution. Halogenation of compounds **2a**-**f** were carried out using the NXS/LiClO_4_/CH_3_CN system (Figure 11).

First, we chlorinated 1,7-bis(3,5-dimethoxyphenyl)-1,6-heptadiene-3,5-dione (**2f**) and determined the structure of product **3f** on the bases of the spectroscopic data and the elemental analysis. In the ^1^H NMR spectrum of **3f**, we observed almost all signals at slightly different chemical shifts as compared with the parent compound **2f**, except the signal at 5.85 ppm for the proton on the C4 position that disappears in **3f**. From mass spectra showing MH^+^ and (MH+2)^+^ signals at m/z = 431 and 433 with a relative intensity of 3:1, we determined that one chlorine atom was present in the molecule. Contrary to the results for 2-chloro-1-phenyl-3-(3,5-dimethoxyphenyl)propane-1,3-dione [34], being in chloroform exclusively in a diketone form, we have found that a α-chloro derivative **3f** is present in *cis* keto-enol form in the chloroform solution (Additional file 2).

We also applied this protocol for bromination of **2f** using NBS as a source of bromine in the presence of LiClO_4_ as catalyst. The reaction was also regioselective and took place exclusively at α-position of 1,3-diketone moiety leading to **4f** that was in *cis* keto-enol form in the CDCl_3_ solution (Additional file 2), as determined by ^1^H, ^13^C NMR and mass spectra.

To establish whether the position of methoxy group in the phenyl ring has any influence on the regioselectively of halogenation, bromination and chlorination, the reactions were carried out with all other isomers **2a**, **2b**; **2c**; **2d** and **2e** (Figure 11).

From the results obtained, we can determine that in all cases bromination and chlorination took place regioselectively on the α-position in the 1,3-diketone moiety and that α-halo products were present dominantly in *cis* keto-enol forms in the CDCl_3_ solution. The conformation of central keto-enol moiety was determined on the basis of ^1^H and ^13^C NMR spectra in CDCl_3_ solution. In the ^13^C NMR spectra, signals for C3 and C5 appear at the same chemical shifts, showing that the enolic fragment of molecules must be symmetric. As in parent compounds, because of rapid tautomerization between both keto-enol forms, average signals for both carbonyl carbon atoms are seen. Due to the effect of halogen atoms bonded at the C4 position, chemical shifts for C3, C5 are a slightly different from those of the corresponding parent compounds (Table 1). In ^1^H NMR spectra, chemical shifts of the enolic proton are higher than in parent compounds, showing that hydrogen-bond strength increases when a halogen atom (chlorine or bromine) is bonded at the C4 position. The slightly different electronic effect of chlorine versus bromine reflects in different OH chemical shifts being for bromo-substituted compounds cca 0.43 ppm higher than for chloro compounds, see Table 1.

**Figure 12:**
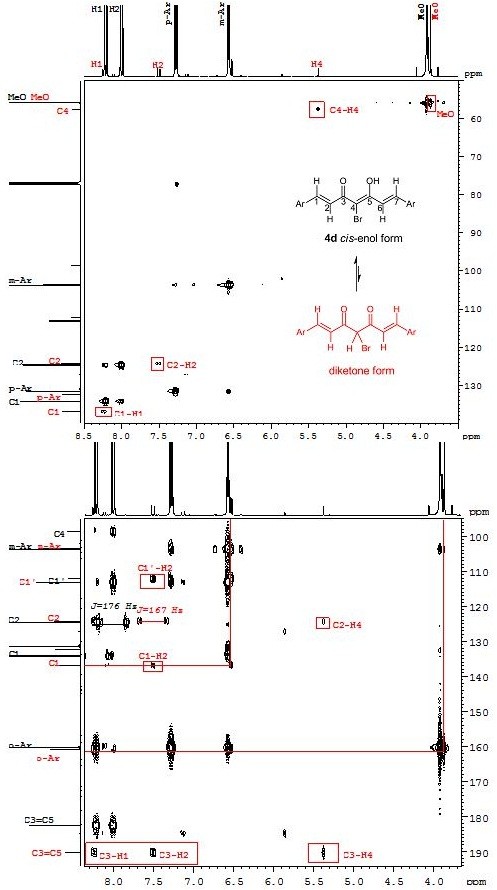
**2D Spectra of 4d.** (Top) Portion of the HSQC spectrum of 4d at 302 K, 500 MHz in CDCl_3_ solution. (Bottom) Portion of the HMBC spectrum.

In some cases, up to 5% of diketone forms were also present in equilibrium in the CDCl_3_ solution. The content of the diketone forms was determined from the ^1^H NMR spectra in which signals for the proton bonded at C4 position were observed. More detailed structure information about the diketone form being in equilibrium with enol form was determined using 2D NMR spectra. For example: in the case of the bromo derivative **4d**, a new signal at 5.37 ppm appears that shows correlation with the signal at 57.5 ppm assigned to C4 in the HSQC spectrum, while in the HMBC spectrum H4 correlates with C2 (at 124.2 ppm) and C3 (190.2 ppm) atoms (Figure 12). The isolate yields and purity of all α-halo compounds are very high and are given in Figure 11.

### Theoretical investigation of some chloro and bromo derivatives

**Figure 13:**
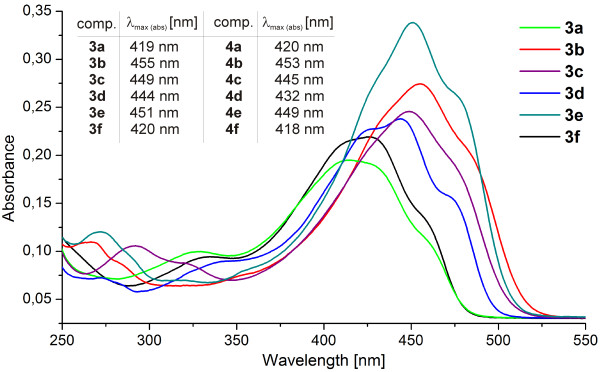
**UV–VIS spectra of compounds 3 compared with tabulated absorption maxima of bromo-derivatives (4) in CH**_**2**_**Cl**_**2 **_**(5.0·10**^**–6 **^**mol/L).**

Comparison of UV–VIS spectra of parent compounds and its 4-chloro and 4-bromo derivatives shows a significant bathochromic shifts, which are in the range of 20–29 nm except in the case of **3d** in which bathochromic shift is higher (39 nm) in the 5.0·10^–6^ M CH_2_Cl_2_ solution (Figure 13).

**Figure 14:**
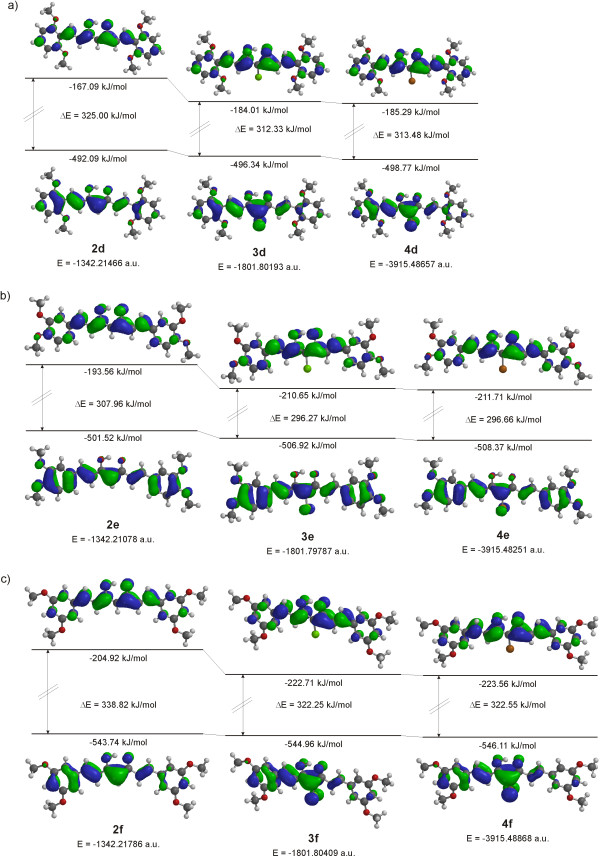
**Molecular orbital diagram levels for HOMOs and LUMOs, computed total energies, and relative energies of compounds. (a)** 2d, 3d, 4d; **(b)** 2e, 3e, 4e and **(c)** 2f, 3f and 4f.

The bonded halogen atoms contribute to the extension of the conjugated system, which is reflected in greater stabilization of the excited state in comparison to the ground state and in lowering of the energy gap between HOMO and LUMO. To confirm the aforementioned experimental statement, theoretical calculation of the completely optimized geometry of 4-chloro and 4-bromo derivatives of 2,6-diOCH_3_, 3,4-diOCH_3_, and 3,5-diOCH_3_ curcuminoids were done. Figure 14 shows the computed energetics, relative energy separations, the molecular orbital energy level diagrams and HOMO-LUMO gaps of enolic forms of corresponding curcuminoids. As seen from the figures, the halogen atoms bonded at position 4 have no influence on the energy of HOMO, while the energy of LUMO is lower in comparison with parent compounds. The HOMO-LUMO gaps are independent from the type of halogen and are nearly the same for the chloro and bromo derivatives. As seen from data for absorption maxima in UV–vis spectra in Figure 13, theoretical calculations are in accord with experimental observation except in the case of **3d**.

**Figure 15:**
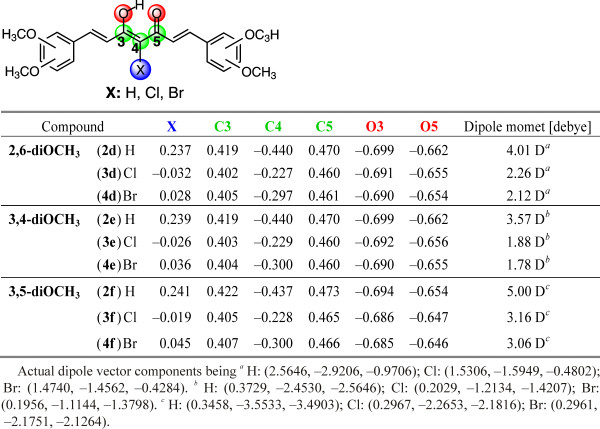
Natural atomic charges for the enolic structure of enolone fragment and dipole moments of 2d–4f.

The participation of electrons of the halogen atom can also be seen from the change of charges at atoms of the enolone part of molecules. In the case of chloro derivatives, small negative charges are present at the chlorine atom: –0.032 in 2,6-diOCH_3_ (**3d**); –0.026 in 3,4-diOCH_3_ (**3e**); –0.019 in 3,5-diOCH_3_ (**3f**) while in bromo derivatives slightly positive charges are present on the bromine atom: 0.028 in 2,6-diOCH_3_ (**4d**); 0.036 in 3,4-diOCH_3_ (**4e**); and 0.045 in 3,5-diOCH_3_ (**4f**) (Figure 15). In contrast, the negative charges on C4 are drastically reduced while slight reductions of negative charges are observed at O3 and O5 atoms, showing calculated electrostatic potential maps of compounds **2e**, **3e** and **4e** in Additional file 1: Figure S3. The positive charges at C3 and C5 are also reduced, see Figure 15. The introduction of halogen atoms at position 4 is also reflected in the planarity of molecules. The dimethoxy substituted phenyl rings bonding at the carbonyl side of molecules are out of plane to a maximum of 5.7 degrees, reflecting a lower level of π-conjugation of these parts of the molecule in comparison with parent compounds (see molecular orbital diagrams for HOMOs in Figure 14).

The influence of halogen atoms at position C4 indicates in changes of total dipole moments being in all cases lower than in parent compounds, see Figure 15.

## Conclusions

In conclusion: all six isomers of dimethoxy curcuminoids (**2a-f**) were prepared using a slight modification of the literature procedures. Higher yields were obtained with no additional purification made with flash chromatography. The structure of single crystal of compounds **2d** and **2e** (polymorphs 1 and 2) were determined by X-ray crystallography. The ORTEP drawing of compound **2d** consists of the asymmetric unit, which contains one 2,6-dimethoxy curcuminoid molecule in *cis* keto-enol form. The solid sample of **2e** contains polymorphs. The crystal structure of polymorph form 1 consists of the symmetric 3,4-dimethoxy curcuminoid molecule in which one half of the molecule is related symmetrically to the other half by a twofold axis. In contrast, the asymmetric unit of polymorph 2 contains two molecules of 3,4-diOCH_3_. ^1^H MAS, ^13^C CPMAS and ^1^H-^13^C 2D HETCOR NMR measurements of solid state of 2,6-diOCH_3_ (**2d**); 3,4-diOCH_3_ (**2e**) and 3,5-diOCH_3_ (**2f**) show that in the case of 2,6-diOCH_3_ only one tautomer is present, while in the case of 3,4-diOCH_3_ and 3,5-diOCH_3_ polymorphism exists. In the CDCl_3_ solution, all compounds **2** are present in forms that can be described as a coexistence of two equivalent tautomers being in fast equilibrium. The position of methoxy groups in compound **2** has little influence on the enolic hydrogen bond and on the position of absorption maximum in UV–VIS spectra. Theoretical calculation for all dimethoxy curcuminoids with complete geometry optimization in the 6-31G(d,p) basis at DFT/B3LYP level of theory for gas phase show that the total energy is different and that the energy gap between HOMO and LUMO are similar for 2,3-diOCH_3_ (**2a**); 2,6-diOCH_3_ (**2d**) and 3,5-diOCH_3_ (**2f**) in one side and for 2,4-diOCH_3_ (**2b**); 2,5-diOCH_3_ (**2c**) and 3,4-diOCH_3_ (**2e**) on the other side. Total energies, dipole moments and HOMO-LUMO gaps for all dimethoxy curcuminoids in CH_2_Cl_2_ solution were also calculated. The total energy stabilization varied from 63.1 to 70.8 kJ/mol, dipole moments are slightly higher in solution than in gas phase while HOMO-LUMO gaps are lower. Calculated UV–VIS spectra are mostly in agreement with the experimental observation in CH_2_Cl_2_ solution (Additional file 1: Figure S1). The low level of participation of electrons from the methoxy groups is reflected in very similar natural atomic charges on oxygen atoms. Chlorination and bromination on α-position of 1,3-diketone moiety of dimethoxy curcumin derivatives did not change the preferred form being cis keto-enol as in the parent compounds. The hydrogen-bond strength increases when chlorine or bromine atom is bonded at the C4 position. 4-Chloro- and 4-bromo derivatives in UV–VIS spectra show significant bathochromic shifts, which are in the range of 20–29 nm except in the case of **3d**. The computed HOMO and LUMO orbitals show that the halogen atom (chlorine or bromine) bonded at C4 have no influence on the energy of HOMO, while the energy of LUMO are lower in comparison with parent compounds. The HOMO-LUMO gaps are independent of the type of halogen. The introduction of halogen atom at C4 is also reflected in the changes of total dipole moments.

## Experimental

### Methods

#### Single-crystal structure analysis

Crystals for X-ray of **2d** and **2e** (polymorph 2) were obtained from a mixture of CH_2_Cl_2_/hexane, followed by slow evaporation in a refrigerator, while single crystals of compound **2e** (polymorph 1) were prepared by recrystallization from CH_3_OH, which was allowed to slowly evaporate at room temperature. Single crystal diffraction data for compounds **2d** and **2e** (polymorph 1) were collected on a Nonius Kappa CCD diffractometer at room temperature with a MoKα radiation and graphite monochromator. The data were processed using the DENZO program. The data for compound **2e** (polymorph 2) have been collected on a Agilent SuperNova dual source diffractometer with an Atlas detector at room temperature with MoKα radiation (0.71073 Å ) and processed using CrysAlis PRO software. All structures were solved with direct methods using SIR97. A full-matrix least-squares refinement on *F*^*2*^ was employed with anisotropic temperature displacement parameters for all non-hydrogen atoms for all compounds. The H atom from enol hydroxy groups and the H atom bonded to *(C4)* atom were located from difference Fourier map in all compounds. These hydrogen atoms were refined freely with their isotropic displacement parameters. In the case of **2e** (polymorph 2) the paremeters of these H atoms were not refiened. The remaining H atoms of all three dimethoxy curcuminoid molecules were placed at idealized, calculated positions and treated as riding, with C–H = 0.93 for C(sp^2^) and 0.96 Å for methyl group and *U*_iso_(H) = 1.2*U*_eq_(C) or 1.5*U*_eq_(C) for C(sp^2^) and methyl group, respectively. Crystals of compound **2e** (polymorph 1) also contain solvent molecules (methanol), which were located in the channels, running parallel *c* axis. These methanol molecules are disordered and consequently their H atoms were not located. The SHELXL97 program was used for structure refinement and interpretation. Drawings of the structures were produced using the ORTEP-3, Platon and Mercury programs. Details of the crystal data, data collection, and refinement parameters are listed in Figures 2, 4 and 5. Structural and other crystallographic data have also been deposited with the Cambridge Crystallographic Data Centre as supplementary publication number CCDC 858801 (**2d**, Additional file 3) & 858802 (**2e** polymorph 1, Additional file 4) & 934715 (**2e** polymorph 2, Additional file 5). A copy of the data can be obtained, free of charge, on application to CCDC, 12 Union Road, Cambridge, CB2 1EZ, UK [fax: +44(0)-1223-336033 or e-mail: deposit@ccdc.cam.ac.uk.

### XRD measurements

X-ray powder diffraction data of solid 1, 2 and 3 were collected using a PANalytical X'Pert PRO (HTK) diffractometer with Cu*K*α radiation. The room temperature reflection data were acquired from 2*Θ* angles of 5° to 30° in steps of 0.034°.

### Solid-state NMR measurements

NMR spectra of solid samples were recorded on a Varian NMR System 600 MHz NMR spectrometer equipped with 3.2 mm NB Double Resonance HX MAS Solids Probe. Larmor frequencies of protons and carbon nuclei were 599.64 MHz and 150.79, respectively. The ^1^H MAS NMR spectra were externally referenced using adamantane. The ^13^C CPMAS NMR spectra were externally referenced using hexamethylbenzene (HMB). All samples were spun at the magic angle with 20 kHz during ^1^H measurement and 16 kHz during ^13^C measurements. Proton spectra were acquired using a spin echo sequence. The repetition delay in all experiments was 5 s. The number of scans was 16. The pulse sequence used for acquiring carbon spectra was a standard cross-polarization MAS pulse sequence with high-power proton decoupling during acquisition. The repetition delay in all experiments was 5 s and the number of scans was between 1000 and 1620. A 2D ^1^H-^13^C HETCOR NMR spectrum was acquired at 16 kHz MAS. Ramp CP transfer with duration of 500 μs was used.

### Theoretical calculation

A theoretical investigation of all species were optimized at the density functional (DFT) level of theory employing the B3LYP/6-31G(d,p) model. Complete geometry optimization was performed for the ground-state structures in the gas phase and CH_2_Cl_2_ solutions of all the molecular systems considered. For the optimized geometries, the Mulliken, natural, and electrostatic atomic charges from the electrostatic potential were calculated. The Spartan'08 suite of quantum chemical programs was used to perform all the calculations, molecular orbital diagrams for HOMOs and LUMOs, the graphic display of the electrostatic potential maps (isovalue = 0.02; property colors range from −90 to 65 kJ/mol ). Theoretical UV–vis spectra were computed by TDDFT/B3LYP/6-31G(d,p) model and visualized using Gaussian function.

### Characterization of compounds

Absorption spectra were recorded with a Lambda 750 UV–vis spectrophotometer from Perkin Elmer. NMR Spectra were recorded at 302 K with a Bruker Avance DPX 300 spectrometer operating at 300 MHz and 75 MHz for ^1^H and ^13^C, and Bruker Avance III 500 spectrometer operating at 500 MHz and 126 MHz for ^1^H and ^13^C, respectively. Proton and carbon spectra were referenced to TMS as the internal standard. Chemical shifts are given on the *δ* scale (ppm). Coupling constants (*J*) are given in Hz. Mass spectra and high-resolution mass spectra were obtained with a Q-TOP Premier instrument. Data are reported as *m/z* (relative intensity). Infrared spectra were recorded on a BIO-RAD Excalibur Series spectrophotometer using samples in potassium bromide disks. Elemental analyses were performed with a Perkin-Elmer 2400 Series II CHNS/O Analyzer. All spectral data obtained for new compounds are reported here. Melting points were measured with a Büchi 535.

### General procedure for 1,7-bis(dimethoxyphenyl)-1,6-heptadiene-3,5-dione

Corresponding dimethoxy benzaldehyde (3.32 g, 20 mmol) was dissolved in ethyl acetate (10–20 mL) and tributylborate (5.94 mL, 22 mmol) was added. The reaction mixure was heated to 75°C. 2,4-Pentanedione (1.03 mL, 10 mmol) and powdery boric anhydride (0.52 g, 7.5 mmol) were mixed separately, carefully heated with stirring to obtain a paste, and added to the mixture of dimethoxybenzaldehyde and tributyl borate. The reaction mixture obtained was heated with stirring for 4.5 hours at 75°C, then cooled to room temperature; *n*-butylamine (0.25 mL, 2.5 mmol) in ethyl acetate (2.5 mL) was then slowly added. Stirring at room temperature continued during the night. The reaction mixture was quenched with the addition of 0.4N HCl (15 mL), heated for 1 hour at 60°C, then cooled on ice, and the solid residue filtered off and washed several times with methanol. The products obtained (**2a**, 2.06 g (52%); **2b**, 2.45 g (62%); **2c**, 2.53 g (64%); **2d**, 2.77 g (70%); **2e**, 1.70 g (43%); **2f**, 1.66 g (42%)) were pure enough to yield satisfactory spectroscopic data (see Additional file 1) and elemental analysis; no additional purification by flash chromatography was necessary.

### General procedure for halogenations

Compound **2** (396 mg, 1 mmol) was dissolved in CH_3_CN (40 mL), LiClO_4_ (64 mg, 0.6 mmol) was added, and the resulting mixture was stirred 10 min at room temperature. An acetonitrile solution of NCS (147 mg, 1.1 mmol) or NBS (196 mg, 1.1 mmol) was slowly added and stirring continued for the corresponding time, given in Figure 11, at room temperature. The product precipitate was filtered off. The filtrate was evaporated and dissolved in methanol. After being refrigerated overnight, an additional quantity of pure product was obtained (in some cases, additional crystallization from the mixture of CH_2_Cl_2_/hexane was done). Overall yields of pure products are given in Figure 11.

#### (1E,6E)-1,7-bis(2,3-dimethoxyphenyl)-4-chloro-1,6-heptadiene-3,5-dione (3a)

Yellow-orange solid, mp 167.1–167.7°C. ^1^H NMR (CDCl_3_, 500 MHz) δ ppm 16.41 (s, –OH), 8.09 (d, 2H, *J* = 15.9), 7.50 (d, 2H, *J* = 15.9), 7.28 (dd, 2H, *J* = 8.0, *J* = 1.0), 7.09 (dd, 2H, *J* = 8.0, *J* = 8.0), 6.97 (dd, 2H, *J* = 8.0, *J* = 1.0), 3.90 (s, 6H), 3.89 (s, 6H). ^13^C NMR (CDCl_3_, 75 MHz) δ ppm 180.5, 153.2, 148.9, 138.1, 129.2, 124.2, 121.2, 119.7, 114.3, 108.5, 61.4, 55.9. IR (KBr) ν = 1617, 1578, 1482, 1428, 1271, 1071, 998, 980, 735 cm^–1^. CIMS (*m*/*z*) 433.1 (MH^+^ +2), 431.1 (MH^+^). CI-HRMS for C_23_H_24_ClO_6_^+^: calcd 431.1261, found 431.1258. EA for C_23_H_23_ClO_6_: calcd 64.11%C, 5.38%H; found 64.31%C, 5.39%H.

#### (1E,6E)-1,7-bis(2,4-dimethoxyphenyl)-4-chloro-1,6-heptadiene-3,5-dione (3b)

Orange solid, mp 169.9–170.8°C. ^1^H NMR (CDCl_3_, 500 MHz) δ ppm 16.78 (s, –OH), 8.04 (d, 2H, *J* = 15.8), 7.58 (d, 2H, *J* = 8.6), 7.40 (d, 2H, *J* = 15.8), 6.54 (dd, 2H, *J* = 8.6, *J* = 2.3), 6.46 (d, 2H, *J* = 2.3), 3.90 (s, 6H), 3.86 (s, 6H). ^13^C NMR (CDCl_3_, 75 MHz) δ ppm 180.6, 163.1, 160.3, 138.2, 130.5, 117.8, 117.4, 107.7, 105.6, 98.3, 55.6, 55.5. IR (KBr) ν = 1596, 1507, 1462, 1298, 1276, 1248, 1212, 1104, 1031, 970, 819 cm^–1^. CIMS (*m*/*z*) 433.1 (MH^+^ +2), 431.1 (MH^+^). CI-HRMS for C_23_H_24_ClO_6_^+^: calcd 431.1261, found 431.1250. EA for C_23_H_23_ClO_6_: calcd 64.11%C, 5.38%H; found 63.80%C, 5.26%H.

#### (1E,6E)-1,7-bis(2,5-dimethoxyphenyl)-4-chloro-1,6-heptadiene-3,5-dione (3c)

Yellow-orange solid, mp 188.2–188.8°C. ^1^H NMR (CDCl_3_, 500 MHz) δ ppm 16.52 (s, –OH), 8.09 (d, 2H, *J* = 15.8), 7.47 (d, 2H, *J* = 15.8), 7.16 (d, 2H, *J* = 3.0), 6.94 (dd, 2H, *J* = 9.0, *J* = 3.0), 6.87 (d, 2H, *J* = 9.0), 3.87 (s, 6H), 3.83 (s, 6H). ^13^C NMR (CDCl_3_, 75 MHz) δ ppm 180.5, 153.6, 153.3, 138.4, 124.7, 120.5, 117.4, 113.5, 112.5, 108.4, 56.2, 55.9. IR (KBr) ν = 1610, 1579, 1496, 1288, 1269, 1053, 1020, 974, 847, 801 cm^–1^. CIMS (*m*/*z*) 433.1 (MH^+^ +2), 431.1 (MH^+^). CI-HRMS for C_23_H_24_ClO_6_^+^: calcd 431.1261, found 431.1262. EA for C_23_H_23_ClO_6_: calcd 64.11%C, 5.38%H; found 63.94%C, 5.25%H.

#### (1E,6E)-1,7-bis(2,6-dimethoxyphenyl)-4-chloro-1,6-heptadiene-3,5-dione (3d)

Yellow-orange solid, mp 159.0–160.3°C. ^1^H NMR (CDCl_3_, 500 MHz) δ ppm 16.77 (s, –OH), 8.23 (d, 2H, *J* = 16.0), 7.91 (d, 2H, *J* = 16.0), 7.28 (dd, 2H, *J* = 8.4, *J* = 8.4), 6.57 (d, 4H, *J* = 8.4), 3.92 (s, 12H). ^13^C NMR (CDCl_3_, 75 MHz) δ ppm 181.6, 160.3, 133.8, 131.5, 122.8, 113.2, 108.5, 103.7, 55.9. IR (KBr) ν = 1603, 1582, 1478, 1258, 1210, 1109, 1035, 735 cm^–1^. CIMS (*m*/*z*) 433.1 (MH^+^ +2), 431.1 (MH^+^). CI-HRMS for C_23_H_24_ClO_6_^+^: calcd 431.1261, found 431.1254. EA for C_23_H_23_ClO_6_: calcd 64.11%C, 5.38%H; found 64.22%C, 5.49%H.

#### (1E,6E)-1,7-bis(3,4-dimethoxyphenyl)-4-chloro-1,6-heptadiene-3,5-dione (3e)

Orange solid, mp 173.8–174.6°C. ^1^H NMR (CDCl_3_, 500 MHz) δ ppm 16.62 (s, –OH), 7.73 (d, 2H, *J* = 15.6), 7.27 (d, 2H, *J* = 15.6), 7.23 (dd, 2H, *J* = 8.3, *J* = 1.8), 7.14 (d, 2H, *J* = 1.8), 6.90 (d, 2H, *J* = 8.3), 3.96 (s, 6H), 3.94 (s, 6H). ^13^C NMR (CDCl_3_, 75 MHz) δ ppm 180.1, 151.5, 149.3, 143.3, 128.1, 123.4, 117.6, 111.2, 110.3, 107.8, 56.0. IR (KBr) ν = 1600, 1512, 1423, 1346, 1263, 1164, 1141, 1024, 969, 804 cm^–1^. CIMS (*m*/*z*) 433.1 (MH^+^ +2), 431.1 (MH^+^). CI-HRMS for C_23_H_24_ClO_6_^+^: calcd 431.1261, found 431.1270. EA for C_23_H_23_ClO_6_: calcd 64.11%C, 5.38%H; found 64.19%C, 5.35%H.

#### (1E,6E)-1,7-bis(3,5-dimethoxyphenyl)-4-chloro-1,6-heptadiene-3,5-dione (3f)

Yellow solid, mp 159.2–159.6°C. ^1^H NMR (CDCl_3_, 500 MHz) δ ppm 16.34 (s, –OH), 7.70 (d, 2H, *J* = 15.6), 7.37 (d, 2H, *J* = 15.6), 6.76 (d, 4H, *J* = 2.1), 6.53 (dd, 2H, *J* = 2.1, *J* = 2.1), 3.84 (s, 12H). ^13^C NMR (CDCl_3_, 75 MHz) δ ppm 180.1, 161.1, 143.5, 136.8, 120.2, 108.4, 106.5, 102.9, 55.5. IR (KBr) ν = 1624, 1596, 1455, 1352, 1290, 1207, 1159, 1062, 966, 835 cm^–1^. CIMS (*m*/*z*) 433.1 (MH^+^ +2), 431.1 (MH^+^). CI-HRMS for C_23_H_24_ClO_6_^+^: calcd 431.1261, found 431.1266. EA for C_23_H_23_ClO_6_: calcd 64.11%C, 5.38%H; found 63.72%C, 5.21%H.

#### (1E,6E)-1,7-bis(2,3-dimethoxyphenyl)-4-bromo-1,6-heptadiene-3,5-dione (4a)

Yellow solid, mp 155.5–155.7°C. ^1^H NMR (CDCl_3_, 500 MHz) δ ppm 16.86 (s, –OH), 8.06 (d, 2H, *J* = 15.8), 7.58 (d, 2H, *J* = 15.8), 7.28 (dd, 2H, *J* = 8.0, *J* = 1.0), 7.09 (dd, 2H, *J* = 8.0, *J* = 8.0), 6.97 (dd, 2H, *J* = 8.0, *J* = 1.0), 3.90 (s, 6H), 3.89 (s, 6H). ^13^C NMR (CDCl_3_, 75 MHz) δ ppm 181.3, 153.2, 148.9, 138.5, 129.1, 124.2, 123.1, 119.8, 114.3, 98.3, 61.4, 55.9. IR (KBr) ν = 1614, 1576, 1482, 1272, 1075, 1000, 975, 742 cm^–1^. CIMS (*m*/*z*) 477.1 (MH^+^ +2), 475.1 (MH^+^). CI-HRMS for C_23_H_24_BrO_6_^+^: calcd 475.0756, found 475.0747. EA for C_23_H_23_BrO_6_: calcd 58.12%C, 4.88%H; found 57.95%C, 4.79%H.

#### (1E,6E)-1,7-bis(2,4-dimethoxyphenyl)-4-bromo-1,6-heptadiene-3,5-dione (4b)

Brown-orange solid, mp 143.1–145.6°C. ^1^H NMR (CDCl_3_, 300 MHz) δ ppm 17.21 (s, –OH), 8.01 (d, 2H, *J* = 15.7), 7.56 (d, 2H, *J* = 8.6), 7.48 (d, 2H, *J* = 15.7), 6.54 (dd, 2H, *J* = 8.6, *J* = 2.3), 6.46 (d, 2H, *J* = 2.3), 3.90 (s, 6H), 3.86 (s, 6H). ^13^C NMR (CDCl_3_, 75 MHz) δ ppm 181.5, 163.1, 160.3, 138.7, 130.7, 119.8, 117.4, 105.6, 98.4, 97.7, 55.6, 55.5. IR (KBr) ν = 1595, 1506, 1464, 1298, 1277, 1252, 1212, 1030, 968, 822 cm^–1^. CIMS (*m*/*z*) 477.1 (MH^+^ +2), 475.1 (MH^+^). CI-HRMS for C_23_H_24_BrO_6_^+^: calcd 475.0756, found 475.0776. EA for C_23_H_23_BrO_6_: calcd 58.12%C, 4.88%H; found 57.73%C, 4.69%H.

#### (1E,6E)-1,7-bis(2,5-dimethoxyphenyl)-4-bromo-1,6-heptadiene-3,5-dione (4c)

Yellow solid, mp 162.9–163.4°C. ^1^H NMR (CDCl_3_, 500 MHz) δ ppm 16.96 (s, –OH), 8.06 (d, 2H, *J* = 15.8), 7.55 (d, 2H, *J* = 15.8), 7.15 (d, 2H, *J* = 3.0), 6.94 (dd, 2H, *J* = 9.0, *J* = 3.0), 6.87 (d, 2H, *J* = 9.0), 3.88 (s, 6H), 3.83 (s, 6H). ^13^C NMR (CDCl_3_, 75 MHz) δ ppm 181.4, 153.5, 153.3, 138.7, 124.6, 122.5, 117.4, 113.7, 112.5, 98.2, 56.2, 55.9. IR (KBr) ν = 1610, 1578, 1497, 1289, 1268, 1053, 1018, 972, 799 cm^–1^. CIMS (*m*/*z*) 477.1 (MH^+^ +2), 475.1 (MH^+^). CI-HRMS for C_23_H_24_BrO_6_^+^: calcd 475.0756, found 475.0760. EA for C_23_H_23_BrO_6_: calcd 58.12%C, 4.88%H; found 57.90%C, 4.80%H.

#### (1E,6E)-1,7-bis(2,6 -dimethoxyphenyl)-4-bromo-1,6-heptadiene-3,5-dione (4d)

Yellow-orange solid, mp 126.7–127.4°C. ^1^H NMR (CDCl_3_, 500 MHz) δ ppm 17.19 (s, –OH), 8.21 (d, 2H, *J* = 15.9), 8.00 (d, 2H, *J* = 15.9), 7.28 (dd, 2H, *J* = 8.4, *J* = 8.4), 6.57 (d, 4H, *J* = 8.4), 3.92 (s, 12H). ^13^C NMR (CDCl_3_, 126 MHz) enol form δ ppm 182.4, 160.3, 134.1, 131.5, 124.6, 113.1, 103.7, 98.6, 55.9; diketone form δ ppm 190.2, 160.7, 136.9, 132.3, 124.2, 112.1, 103.6, 57.5, 55.8. IR (KBr) ν = 1603, 1580, 1477, 1397, 1257, 1207, 1110, 1036, 737cm^–1^. CIMS (*m*/*z*) 477.1 (MH^+^ +2), 475.1 (MH^+^). CI-HRMS for C_23_H_24_BrO_6_^+^: calcd 475.0756, found 475.0752. EA for C_23_H_23_BrO_6_: calcd 58.12%C, 4.88%H; found 58.21%C, 4.78%H.

#### (1E,6E)-1,7-bis(3,4-dimethoxyphenyl)-4-bromo-1,6-heptadiene-3,5-dione (4e)

Orange solid mp, 160.9–161.3°C. ^1^H NMR (CDCl_3_, 500 MHz) δ ppm 17.05 (s, –OH), 7.72 (d, 2H, *J* = 15.5), 7.34 (d, 2H, *J* = 15.5), 7.23 (dd, 2H, *J* = 8.3, *J* = 1.9), 7.13 (d, 2H, *J* = 1.9), 6.90 (d, 2H, *J* = 8.3), 3.96 (s, 6H), 3.94 (s, 6H). ^13^C NMR (CDCl_3_, 75 MHz) δ ppm 181.0, 151.5, 149.3, 143.7, 128.0, 123.3, 119.4, 111.2, 110.3, 97.6, 56.0. IR (KBr) ν = 1599, 1512, 1422, 1343, 1261, 1236, 1163, 1140, 1025, 967, 806 cm^–1^. CIMS (*m*/*z*) 477.1 (MH^+^ +2), 475.1 (MH^+^). CI-HRMS for C_23_H_24_BrO_6_^+^: calcd 475.0756, found 475.0750. EA for C_23_H_23_BrO_6_: calcd 58.12%C, 4.88%H; found 58.31%C, 4.86%H.

#### (1E,6E)-1,7-bis(3,5-dimethoxyphenyl)-4-bromo-1,6-heptadiene-3,5-dione (4f)

Yellow solid, mp 154.7–154.9°C. ^1^H NMR (CDCl_3_, 500 MHz) δ ppm 16.79 (s, –OH), 7.68 (d, 2H, *J* = 15.6), 7.44 (d, 2H, *J* = 15.6), 6.76 (d, 4H, *J* = 2.2), 6.53 (dd, 2H, *J* = 2.2, *J* = 2.2), 3.84 (s, 12H). ^13^C NMR (CDCl_3_, 75 MHz) δ ppm 181.0, 161.1, 143.8, 136.8, 122.1, 106.5, 102.8, 98.1, 55.5. IR (KBr) ν = 1621, 1595, 1461, 1299, 1209, 1157, 1063, 967, 835 cm^–1^. CIMS (*m*/*z*) 477.1 (MH^+^ +2), 475.1 (MH^+^). CI-HRMS for C_23_H_24_BrO_6_^+^: calcd 475.0756, found 475.0752. EA for C_23_H_23_BrO_6_: calcd 58.12%C, 4.88%H; found 57.95%C, 4.66%H.

## Competing interests

The authors declare that they have no competing interests.

## Authors’ contributions

PG all the experimental work, the spectroscopic characterization, helped to draft the manuscript; AG the single-crystal structure analysis; JK participated in the theoretical calculation; BK participated in the theoretical calculation and designed the manuscript; BŠ draft the manuscript. All the authors read and approved the final manuscript.

## Supplementary Material

Additional file 1 Supporting file. Click here for file

Additional file 2 Copies of the Spectra. Click here for file

Additional file 3 deposit1. Click here for file

Additional file 4 deposit2. Click here for file

Additional file 5 mo4kon. Click here for file
