# Supplementary material for: Structures in solid state and solution of dimethoxy curcuminoids: regioselective bromination and chlorination
Source: Chem Cent J. 2013 Jun 25;7:107. doi: 10.1186/1752-153X-7-107 (PMC3720230; doi:10.1186/1752-153X-7-107)
Supplement: Additional file 1 — Supporting file. [file 1752-153X-7-107-S1.doc]

# Structures in solid state and solution of dimethoxy curcuminoids: Regioselective bromination and chlorination

Petra Galer^§^, Amalija Golobič, Jože Koller, Berta Košmrlj and Boris Šket*

Faculty of Chemistry and Chemical Technology, University of Ljubljana, Aškerčeva 5, SI-1000 Ljubljana, Slovenia

Email addresses:

Petra Galer: [petra.galer@fkkt.uni-lj.si](mailto:petra.galer@fkkt.uni-lj.si)

Amalija Golobič: [malci.golobic@fkkt.uni-lj.si](mailto:malci.golobic@fkkt.uni-lj.si)

Jože Koller: [joze.koller@fkkt.uni-lj.si](mailto:joze.koller@fkkt.uni-lj.si)

Berta Košmrlj: [berta.kosmrlj@fkkt.uni-lj.si](mailto:berta.kosmrlj@fkkt.uni-lj.si)

Boris Šket: [boris.sket@fkkt.uni-lj.si](mailto:boris.sket@fkkt.uni-lj.si)

**Table of contents:**

Experimental data of compounds **2a-f** 2

Figure S1 Calculated UV-Vis spectra of **2a-f** 4

Figure S2 XRD patterns and Solid-state NMR spectra of **2e**, solid 3 5

Figure S3 Electrostatic-potential maps of **2e**, **3e** and **4e** 6

Figure S4 X-ray structure determination of **2e**, polymorph 1 7

## Experimental data of compounds 2a-f

## (1E,6E)-1,7-bis(2,3-dimethoxyphenyl)-1,6-heptadiene-3,5-dione (2a) [[[1]](#footnote-2)]:

Intense yellow solid, mp 118.7–119.6 °C (lit. 117–120 °C). ^1^H NMR (CDCl_3_, 300 MHz) δ ppm 15.91 (br s, –OH), 7.97 (d, 2H, *J* = 16.1), 7.20 (dd, 2H, *J* = 7.9, *J* = 1.0), 7.07 (dd, 2H, *J* = 8.0, *J* = 7.9), 6.94 (dd, 2H, *J* = 8.0, *J* = 1.0), 6.70 (d, 2H, *J* = 16.1), 5.90 (s, 1H), 3.89 (s, 12H). ^13^C NMR (CDCl_3_, 75 MHz) δ ppm 183.6, 153.2, 148.6, 135.2, 129.2, 125.6, 124.1, 119.2, 113.8, 101.6, 61.3, 55.9. IR (KBr) ν = 1623, 1577, 1481, 1429, 1266, 1138, 1070, 1000, 739cm^–1^.

## (1E,6E)-1,7-bis(2,4-dimethoxyphenyl)-1,6-heptadiene-3,5-dione (2b) [[[2]](#footnote-3)]:

Brown-orange solid, mp 145.2–146.3 °C (lit. 135–137 °C). ^1^H NMR (CDCl_3_, 300 MHz) δ ppm 16.19 (br s, –OH), 7.89 (d, 2H, *J* = 15.9), 7.48 (d, 2H, *J* = 8.6), 6.61 (d, 2H, *J* = 15.9), 6.51 (dd, 2H, *J* = 8.6, *J* = 2.3), 6.45 (d, 2H, *J* = 2.3), 5.80 (s, 1H), 3.88 (s, 6H), 3.84 (s, 6H). ^13^C NMR (CDCl_3_, 75 MHz) δ ppm 183.8, 162.6, 159.9, 135.4, 130.1, 122.5, 117.4, 105.4, 101.1, 98.4, 55.5, 55.5. IR (KBr) ν = 1612, 1460, 1276, 1211, 1162, 1033, 971, 822 cm^–1^.

## (1E,6E)-1,7-bis(2,5-dimethoxyphenyl)-1,6-heptadiene-3,5-dione (2c) [[[3]](#footnote-4)]:

Brick-red solid, mp 117.9–120.2 °C (lit. 119–122 °C). 1H NMR (CDCl3, 300 MHz) δ ppm 15.96 (br s, –OH), 7.95 (d, 2H, *J* = 16.0), 7.09 (d, 2H, *J* = 2.8), 6.91 (dd, 2H, *J* = 9.0, *J* = 2.8), 6.85 (d, 2H, *J* = 9.0), 6.69 (d, 2H, *J* = 16.0), 5.89 (s, 1H), 3.86 (s, 6H), 3.81 (s, 6H). ^13^C NMR (CDCl_3_, 75 MHz) δ ppm 183.6, 153.6, 153.0, 135.5, 125.0, 124.7, 116.9, 113.1, 112.5, 101.5, 56.1, 55.8. IR (KBr) ν = 1621, 1495, 1288, 1220, 1137, 1048, 1026, 973, 799 cm^–1^.

## (1E,6E)-1,7-bis(2,6-dimethoxyphenyl)-1,6-heptadiene-3,5-dione (2d) [3]:

Orange solid, mp 185.6–186.2 °C (lit. 180–184 °C). ^1^H NMR (CDCl_3_, 300 MHz) δ ppm 16.23 (br s, –OH), 8.11 (d, 2H, *J* = 16.2), 7.25 (dd, 2H, *J* = 8.4, *J* = 8.4), 7.12 (d, 2H, *J* = 16.2), 6.56 (d, 4H, *J* = 8.4), 5.85 (s, 1H), 3.89 (s, 12H). ^13^C NMR (CDCl_3_, 75 MHz) δ ppm 184.7, 160.0, 131.1, 130.9, 127.2, 113.1, 103.8, 102.0, 55.8. IR (KBr) ν = 1615, 1583, 1476, 1257, 1112, 1037, 986, 768 cm^–1^.

## (1E,6E)-1,7-bis(3,4-dimethoxyphenyl)-1,6-heptadiene-3,5-dione (2e) [[[4]](#footnote-5)]:

Orange solid, mp 129.2–130.1 °C (lit. 128–130 °C). ^1^H NMR (CDCl_3_, 300 MHz) δ ppm 16.02 (br s, –OH), 7.61 (d, 2H, *J* = 15.8), 7.15 (dd, 2H, *J* = 8.3, *J* = 1.9), 7.08 (d, 2H, *J* = 1.9), 6.89 (d, 2H, *J* = 8.3), 6.50 (d, 2H, *J* = 15.8), 5.83 (s, 1H), 3.94 (s, 6H), 3.93 (s, 6H). ^13^C NMR (CDCl_3_, 75 MHz) δ ppm 183.2, 151.1, 149.3, 140.4, 128.1, 122.6, 122.1, 111.2, 109.8, 101.2, 56.0, 55.9. IR (KBr) ν = 1624, 1583, 1513, 1463, 1263, 1136, 1024, 967, 808 cm^–1^.

## (1E,6E)-1,7-bis(3,5-dimethoxyphenyl)-1,6-heptadiene-3,5-dione (2f) [[[5]](#footnote-6)]:

Intense yellow solid, mp 144.9–145.5 °C (lit. 139–142 °C). ^1^H NMR (CDCl_3_, 300 MHz) δ ppm 15.83 (br s, –OH), 7.57 (d, 2H, *J* = 15.8), 6.69 (d, 4H, *J* = 2.2), 6.58 (d, 2H, *J* = 15.8), 6.49 (dd, 2H, *J* = 2.2, *J* = 2.2), 5.85 (s, 1H), 3.82 (s, 12H). ^13^C NMR (CDCl_3_, 75 MHz) δ ppm 183.2, 161.0, 140.6, 136.9, 124.6, 106.0, 102.4, 101.8, 55.4. IR (KBr) ν = 1636, 1595, 1456, 1302, 1206, 1155, 1065, 967, 833 cm^–1^.

## Calculated UV-Vis spectra of compounds 2a-f


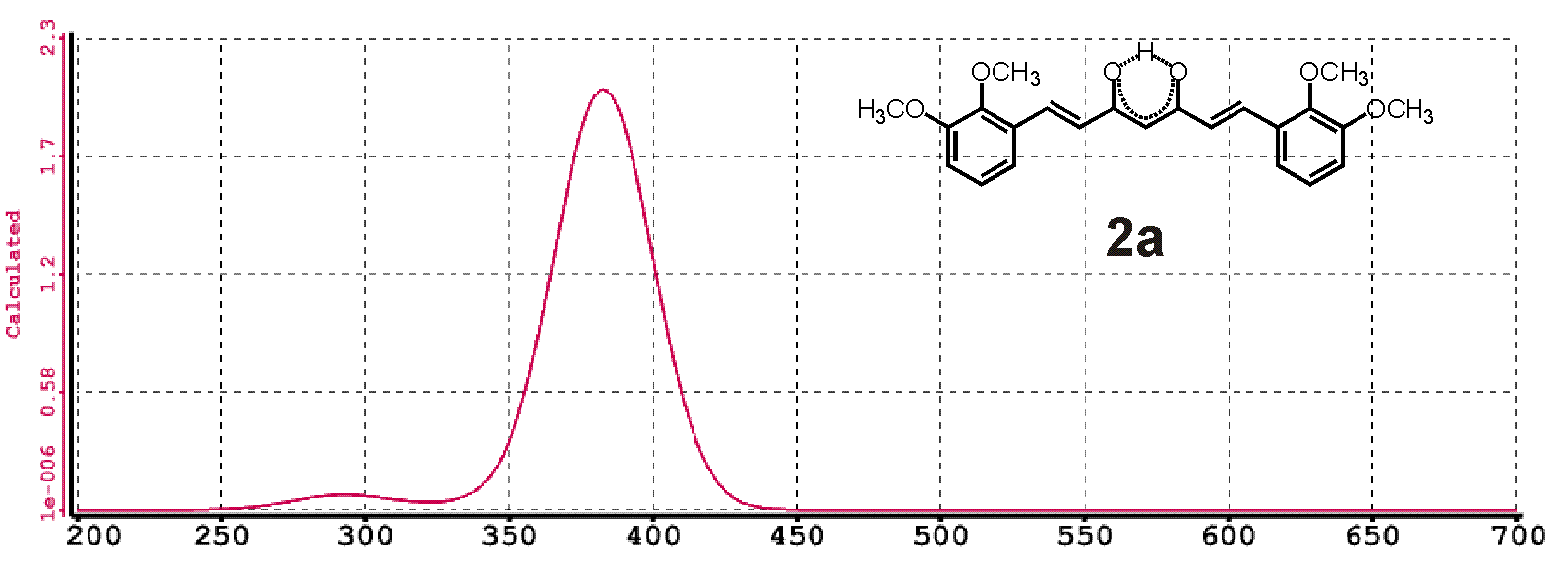


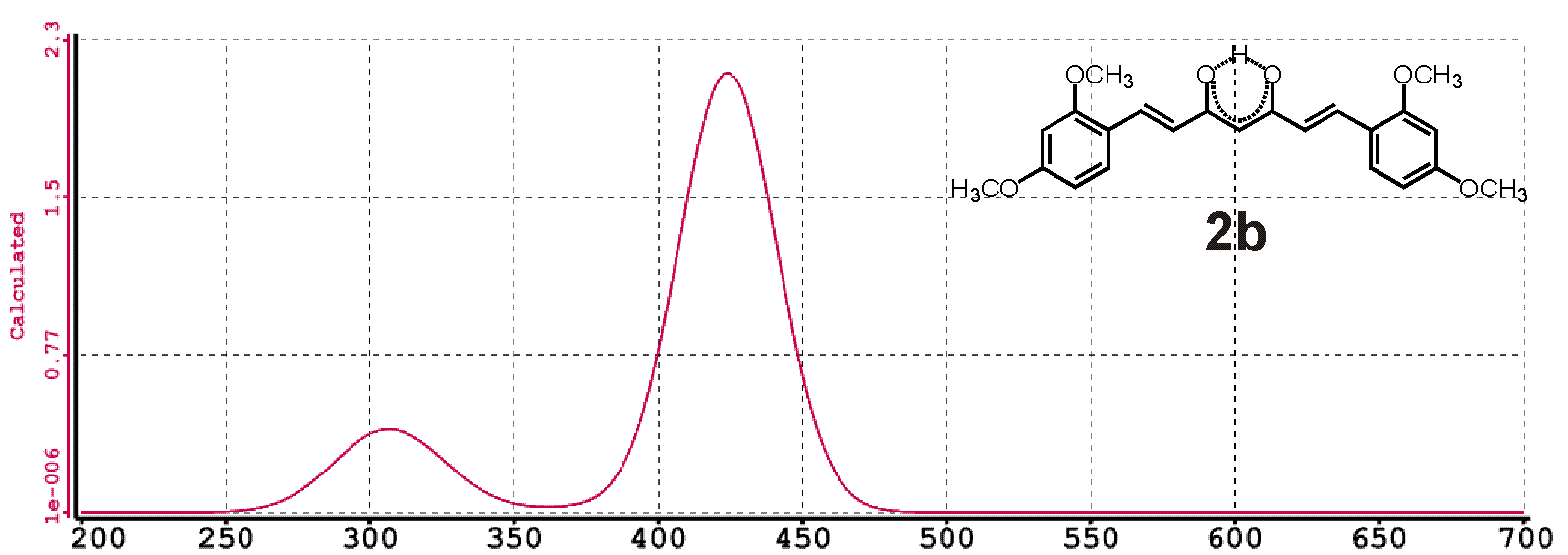


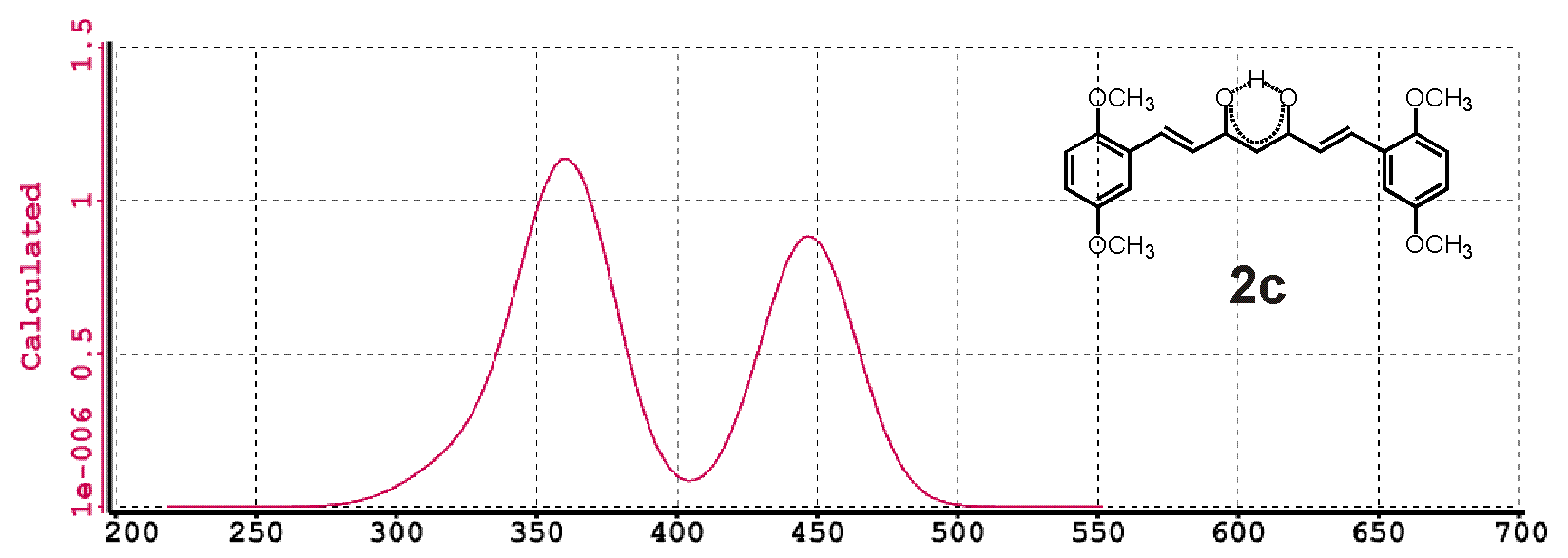


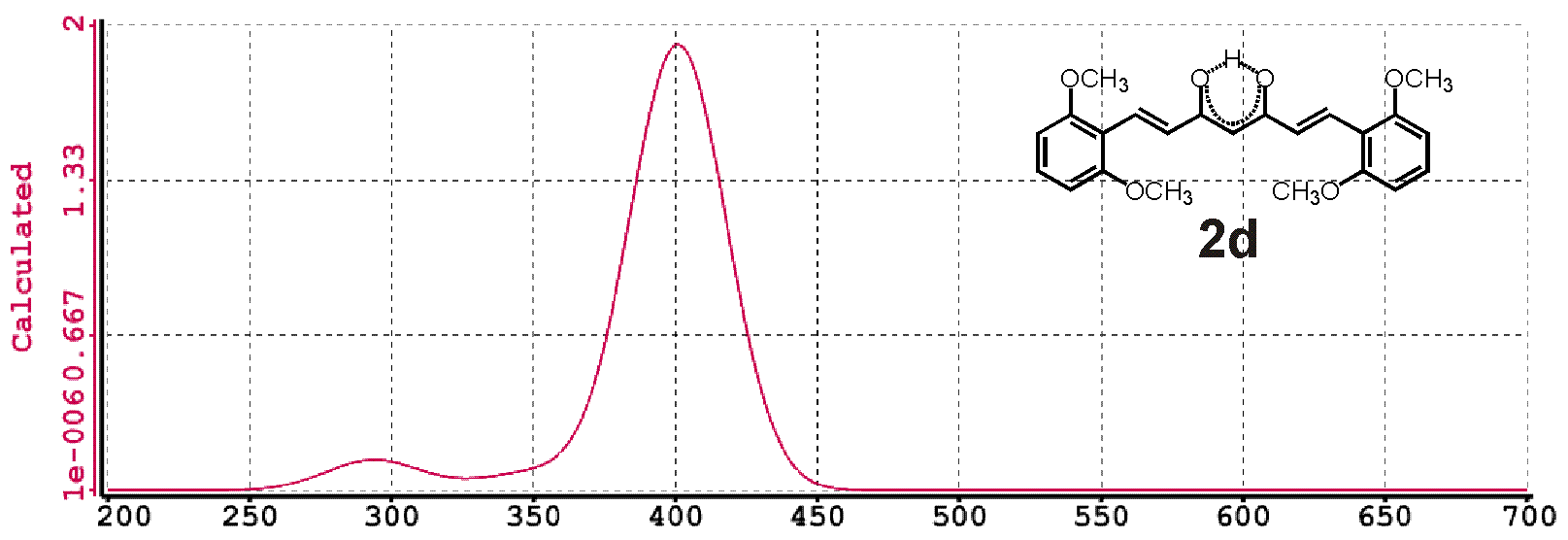


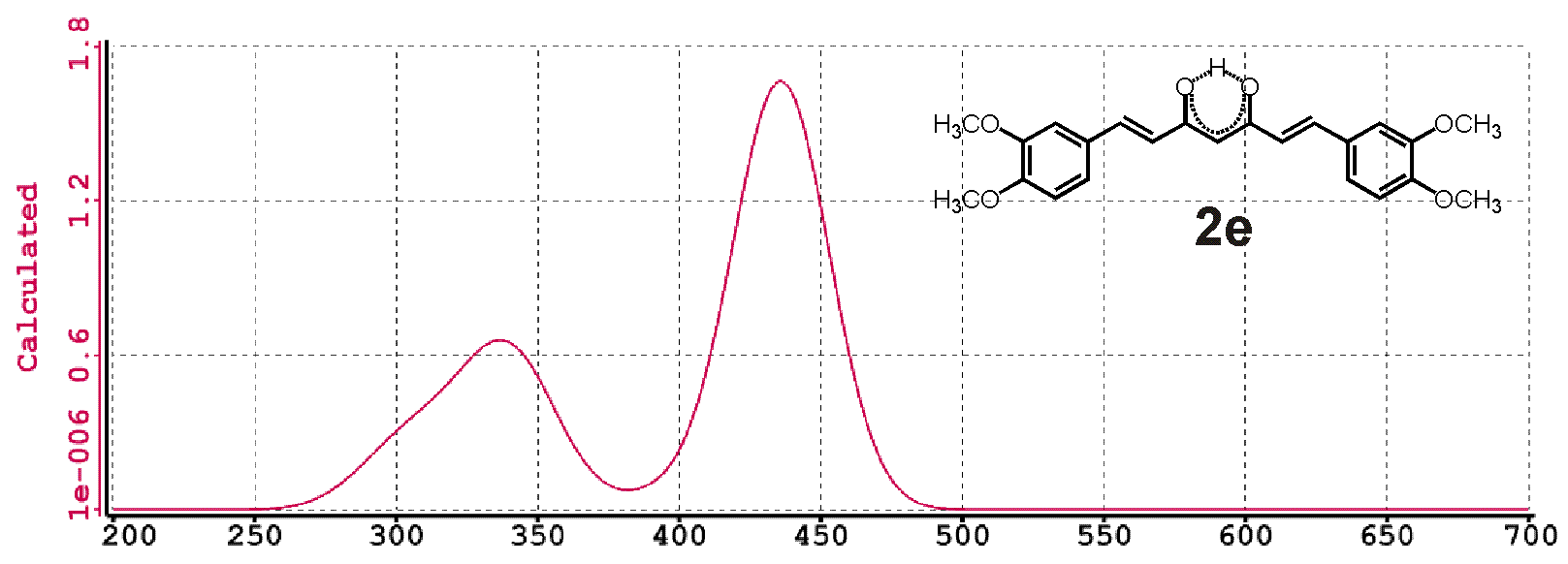


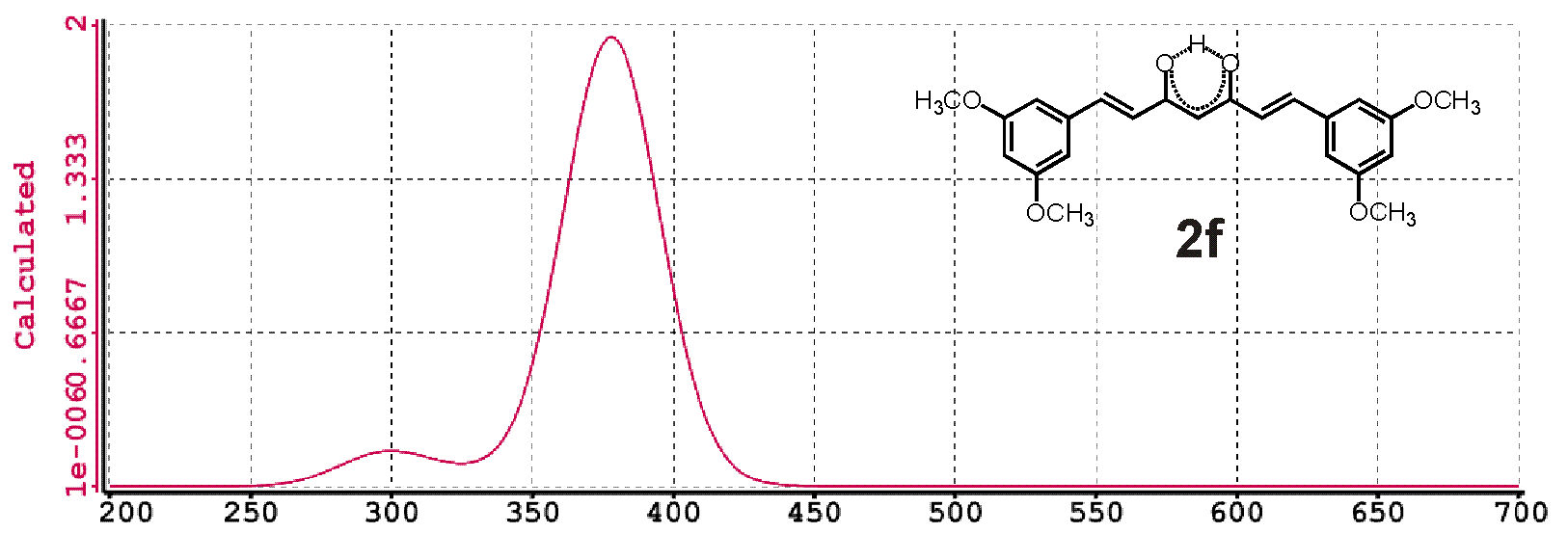


**Figure S1** Simulated UV-Vis spectra of compounds **2a**-**f** in CH_2_Cl_2_ obtained by TDDFT/B3LYP 631-G(d,p) basis sets and visualized by Gaussian function.


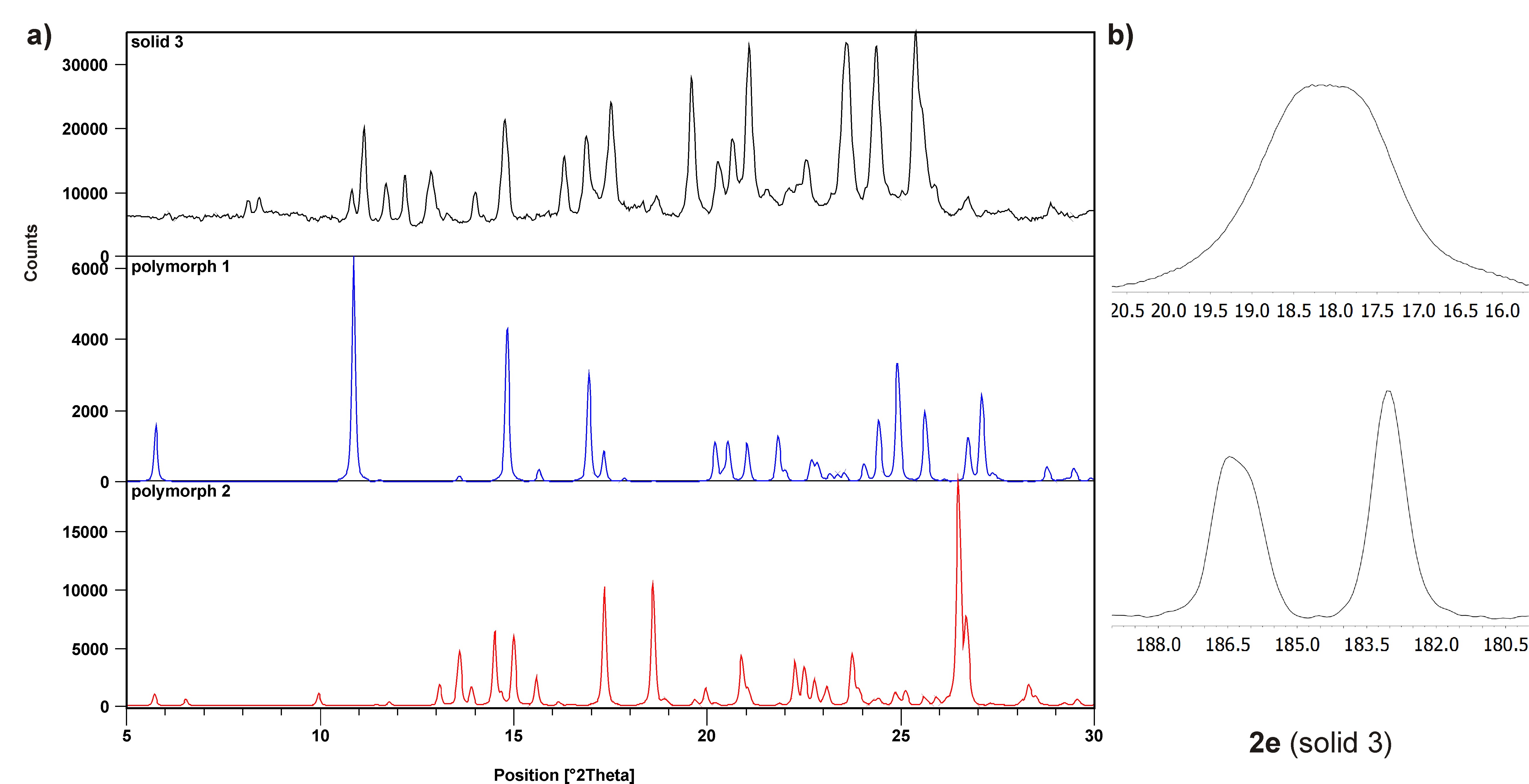


## Figure S2 a) XRD diagrams. Measured pattern of 2e (solid 3) and calculated pattern of polymorph 1 and polymorph 2. b) ^1^H echo MAS NMR spectra (up) and ^1^H-^13^C CPMAS NMR spectra (down).


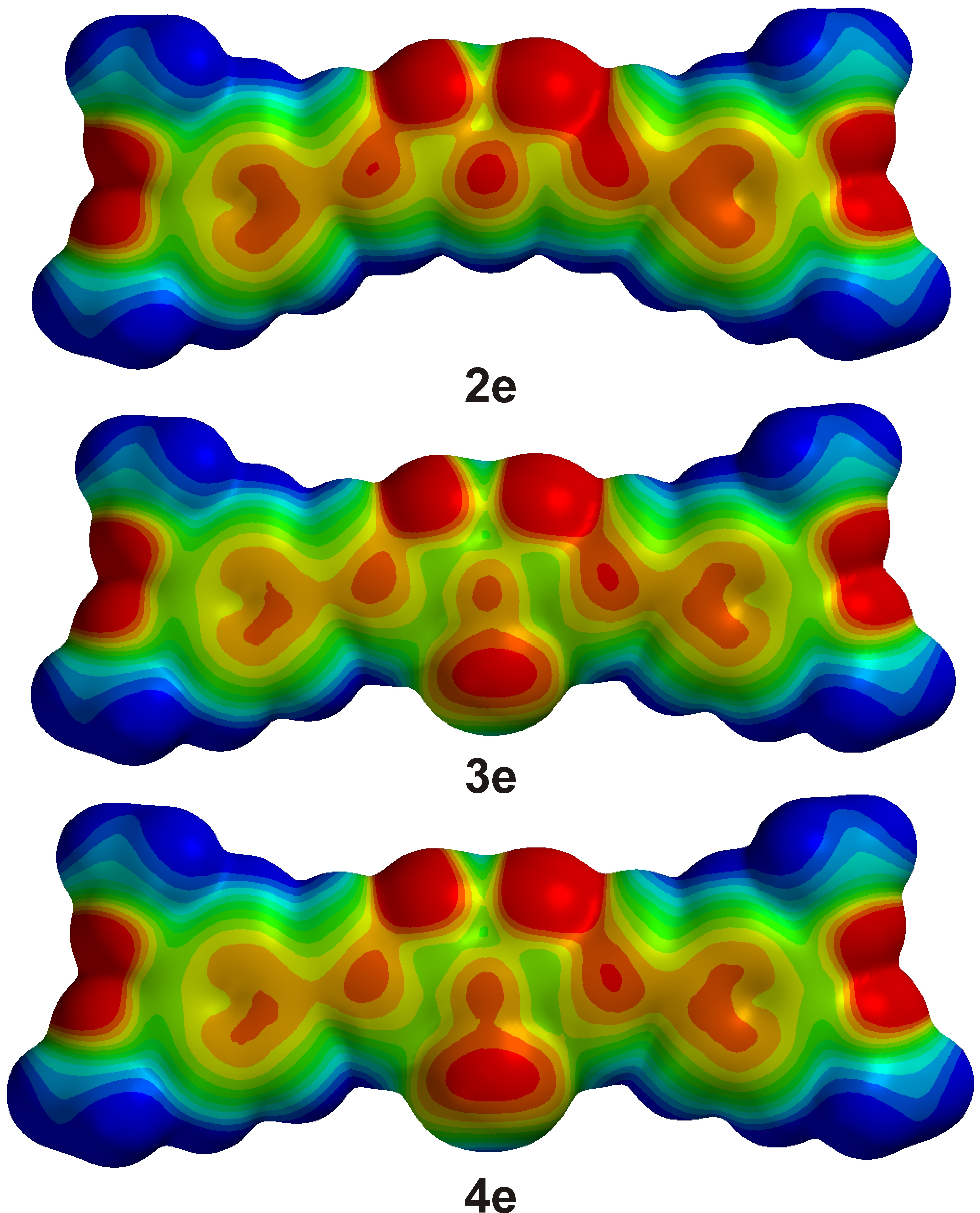


## Figure S3 Electrostatic-potential maps clearly show the difference in the electrostatic nature on C4 position between 2e and its parent chloro (3e) and bromo (4e) analogue. The colours indicate the value of the electrostatic potential. Colors near red correspond to areas on the surface that are negatively charged (attracted to a point-positive charge), while colors toward blue designate regions that are positively charged. Intermediate colors (greens) represent neutral regions.


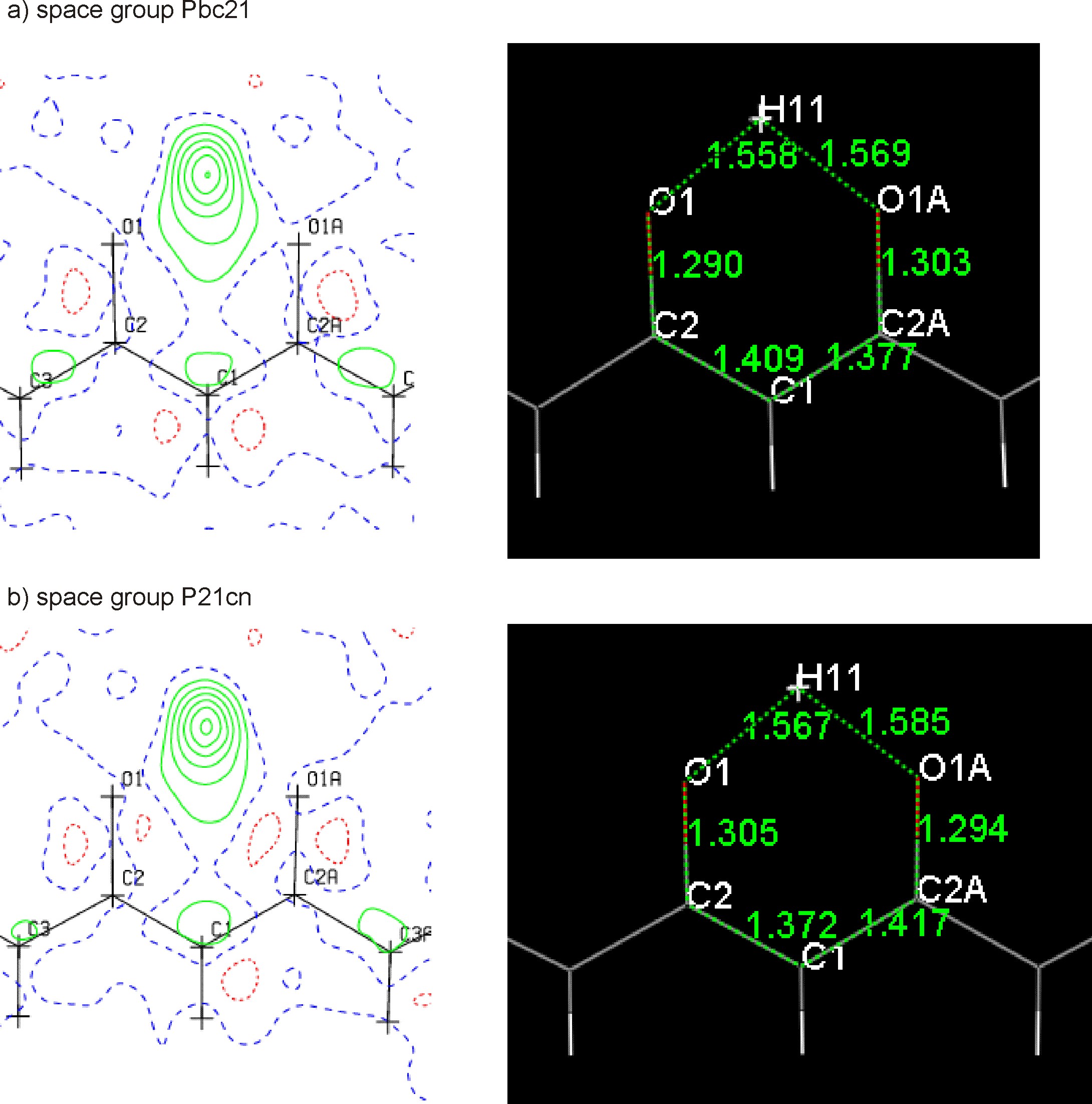


**Figure S4** X-ray structure determination of compound **2e** (polymorph **1**) was performed also in space groups Pbc21 (no.29) and P21cn (no.33) which are subgroups (without two fold axis) of final space group Pbcn (no. 60). In both cases the asymmetric unit contains whole molecule (and not half of it as in the final solution). In both cases bond distances showed delocalized structure and a difference Fourier map resulted to a maximum for H-atom centred between both O atoms (the obtained geometrical parameters were very close to twofold symmetry of molecules). Because there were no significant differences in geometry of both halves of molecules and also no better agreement factors of the structure analysis (Rfactors , goodnes of fit....) we took as final result structure solution in more symmetric space group Pbcn (no. 60).

1. Weber WM, Hunsaker LA, Abcouwer SF, Deck LM, Jagt DLV (2005): **Anti-Oxidant Activities of Curcumin and Related Enones.** *Bioorg. Med. Chem*, **13**: 3811–3820. [↑](#footnote-ref-2)
2. Lin L, Shi Q, Nyarko AK, Bastow KF, Wu C-C, Su C-Y, Shih CC-Y, Lee K-H (2006): **Antitumor Agents. 250. Design and Synthesis of New Curcumin Analogues as Potential Anti-Prostate Cancer Agents.** *J. Med. Chem*, **49**: 3963–3972. [↑](#footnote-ref-3)
3. Takahashi T, Hijikuro I, Sugimoto H, Kihara T, Shimmyo Y, Niidome T (2008): **Novel Curcumin Derivative.** WO 2008066151 (A1). [↑](#footnote-ref-4)
4. Nurfina AN, Reksohadiprodjo MS, Timmerman H, Jenie UA, Sugiyanto D, van der Goot H (1997): **Synthesis of some Symmetrical Curcumin Derivatives and their Antiinflammatory Activity.** *Eur. J. Med. Chem*, **32**: 321–328. [↑](#footnote-ref-5)
5. Hahm E-R, Cheon G, Lee J, Kim B, Park C, Yang C-H (2002): **New and Known Symmetrical Curcumin Derivatives Inhibit the Formation of Fos-Jun-DNA Complex.** *Cancer Lett*, **184**: 89–96. [↑](#footnote-ref-6)
