# Supplementary material for: Structures in solid state and solution of dimethoxy curcuminoids: regioselective bromination and chlorination
Source: Chem Cent J. 2013 Jun 25;7:107. doi: 10.1186/1752-153X-7-107 (PMC3720230; doi:10.1186/1752-153X-7-107)
Supplement: Additional file 2 — Copies of the Spectra. [file 1752-153X-7-107-S2.doc]

# Structures in solid state and solution of dimethoxy curcuminoids: Regioselective bromination and chlorination

Petra Galer^§^, Amalija Golobič, Jože Koller, Berta Košmrlj and Boris Šket*

Faculty of Chemistry and Chemical Technology, University of Ljubljana, Askerceva 5, SI-1000 Ljubljana, Slovenia

Email addresses:

Petra Galer: [petra.galer@fkkt.uni-lj.si](mailto:petra.galer@fkkt.uni-lj.si)

Amalija Golobič: [malci.golobic@fkkt.uni-lj.si](mailto:malci.golobic@fkkt.uni-lj.si)

Jože Koller: [joze.koller@fkkt.uni-lj.si](mailto:joze.koller@fkkt.uni-lj.si)

Berta Košmrlj: [berta.kosmrlj@fkkt.uni-lj.si](mailto:berta.kosmrlj@fkkt.uni-lj.si)

Boris Šket: [boris.sket@fkkt.uni-lj.si](mailto:boris.sket@fkkt.uni-lj.si)

**Table of contents:**

**^1^H and ^13^C NMR spectra of:**

(1E,6E)-1,7-bis(2,3-dimethoxyphenyl)-1,6-heptadiene-3,5-dione (**2a**) 2

(1E,6E)-1,7-bis(2,4-dimethoxyphenyl)-1,6-heptadiene-3,5-dione (**2b**) 2

(1E,6E)-1,7-bis(2,5-dimethoxyphenyl)-1,6-heptadiene-3,5-dione (**2c**) 3

(1E,6E)-1,7-bis(2,6-dimethoxyphenyl)-1,6-heptadiene-3,5-dione (**2d**) 3

(1E,6E)-1,7-bis(3,4-dimethoxyphenyl)-1,6-heptadiene-3,5-dione (**2e**) 4

(1E,6E)-1,7-bis(3,5-dimethoxyphenyl)-1,6-heptadiene-3,5-dione (**2f**) 4

(1E,6E)-1,7-bis(2,3-dimethoxyphenyl)-4-chloro-1,6-heptadiene-3,5-dione (**3a**) 5

(1E,6E)-1,7-bis(2,4-dimethoxyphenyl)-4-chloro-1,6-heptadiene-3,5-dione (**3b**) 5

(1E,6E)-1,7-bis(2,5-dimethoxyphenyl)-4-chloro-1,6-heptadiene-3,5-dione (**3c**) 6

(1E,6E)-1,7-bis(2,6-dimethoxyphenyl)-4-chloro-1,6-heptadiene-3,5-dione (**3d**) 6

(1E,6E)-1,7-bis(3,4-dimethoxyphenyl)-4-chloro-1,6-heptadiene-3,5-dione (**3e**) 7

(1E,6E)-1,7-bis(3,5-dimethoxyphenyl)-4-chloro-1,6-heptadiene-3,5-dione (**3f**) 7

(1E,6E)-1,7-bis(2,3-dimethoxyphenyl)-4-bromo-1,6-heptadiene-3,5-dione (**4a**) 8

(1E,6E)-1,7-bis(2,4-dimethoxyphenyl)-4-bromo-1,6-heptadiene-3,5-dione (**4b**) 8

(1E,6E)-1,7-bis(2,5-dimethoxyphenyl)-4-bromo-1,6-heptadiene-3,5-dione (**4c**) 9

(1E,6E)-1,7-bis(2,6 -dimethoxyphenyl)-4-bromo-1,6-heptadiene-3,5-dione (**4d**) 9

(1E,6E)-1,7-bis(3,4-dimethoxyphenyl)-4-bromo-1,6-heptadiene-3,5-dione (**4e**) 10

(1E,6E)-1,7-bis(3,5-dimethoxyphenyl)-4-bromo-1,6-heptadiene-3,5-dione (**4f**) 10

(1E,6E)-1,7-bis(2,3-dimethoxyphenyl)-1,6-heptadiene-3,5-dione (**2a**)

(1E,6E)-1,7-bis(2,4-dimethoxyphenyl)-1,6-heptadiene-3,5-dione (**2b**)

(1E,6E)-1,7-bis(2,5-dimethoxyphenyl)-1,6-heptadiene-3,5-dione (**2c**)

(1E,6E)-1,7-bis(2,6-dimethoxyphenyl)-1,6-heptadiene-3,5-dione (**2d**)

(1E,6E)-1,7-bis(3,4-dimethoxyphenyl)-1,6-heptadiene-3,5-dione (**2e**)

(1E,6E)-1,7-bis(3,5-dimethoxyphenyl)-1,6-heptadiene-3,5-dione (**2f**)

(1E,6E)-1,7-bis(2,3-dimethoxyphenyl)-4-chloro-1,6-heptadiene-3,5-dione (**3a**)

(1E,6E)-1,7-bis(2,4-dimethoxyphenyl)-4-chloro-1,6-heptadiene-3,5-dione (**3b**)


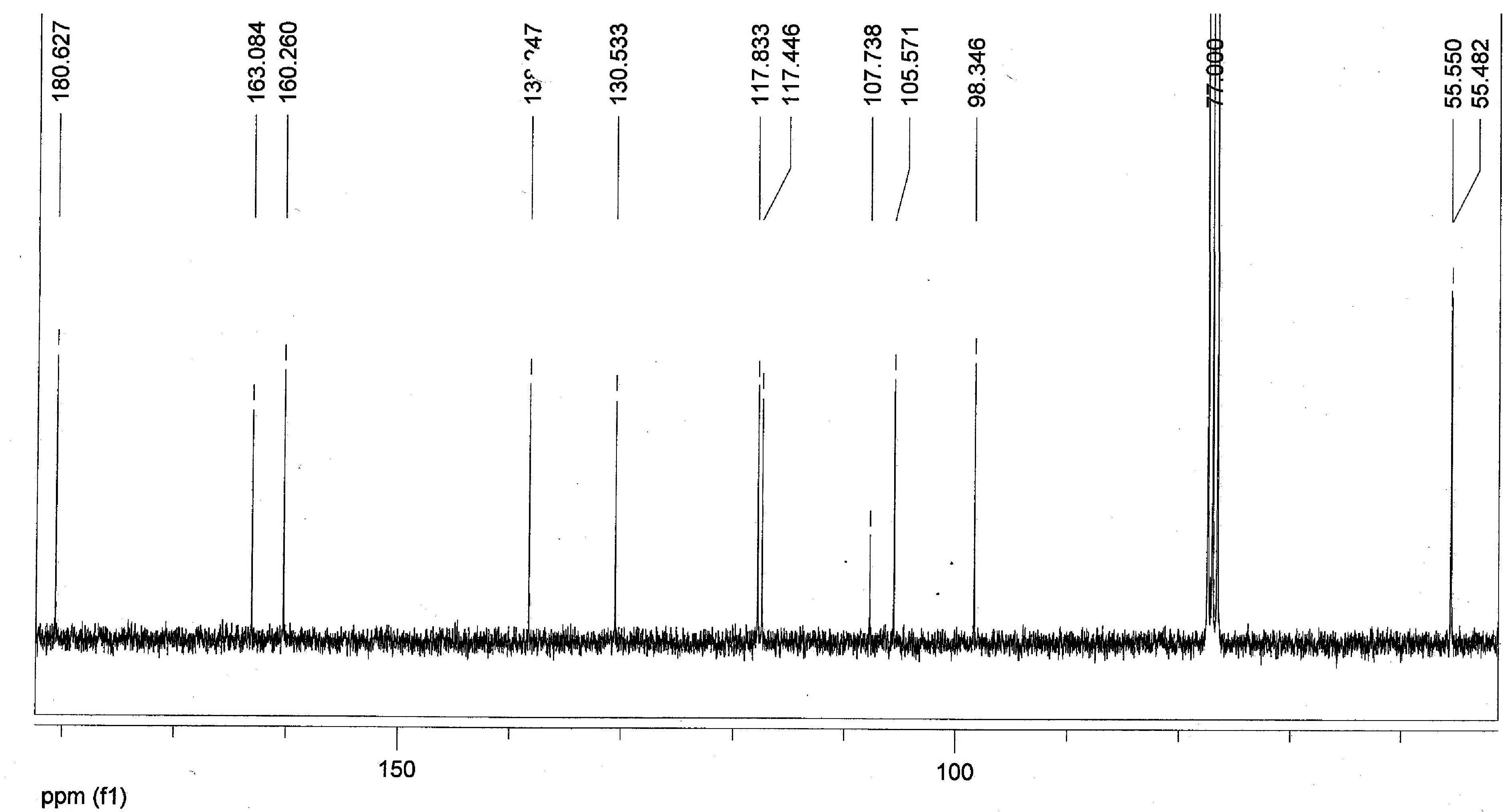


(1E,6E)-1,7-bis(2,5-dimethoxyphenyl)-4-chloro-1,6-heptadiene-3,5-dione (**3c**)

(1E,6E)-1,7-bis(2,6-dimethoxyphenyl)-4-chloro-1,6-heptadiene-3,5-dione (**3d**)

(1E,6E)-1,7-bis(3,4-dimethoxyphenyl)-4-chloro-1,6-heptadiene-3,5-dione (**3e**)

(1E,6E)-1,7-bis(3,5-dimethoxyphenyl)-4-chloro-1,6-heptadiene-3,5-dione (**3f**)

(1E,6E)-1,7-bis(2,3-dimethoxyphenyl)-4-bromo-1,6-heptadiene-3,5-dione (**4a**)

(1E,6E)-1,7-bis(2,4-dimethoxyphenyl)-4-bromo-1,6-heptadiene-3,5-dione (**4b**)

(1E,6E)-1,7-bis(2,5-dimethoxyphenyl)-4-bromo-1,6-heptadiene-3,5-dione (**4c**)

**
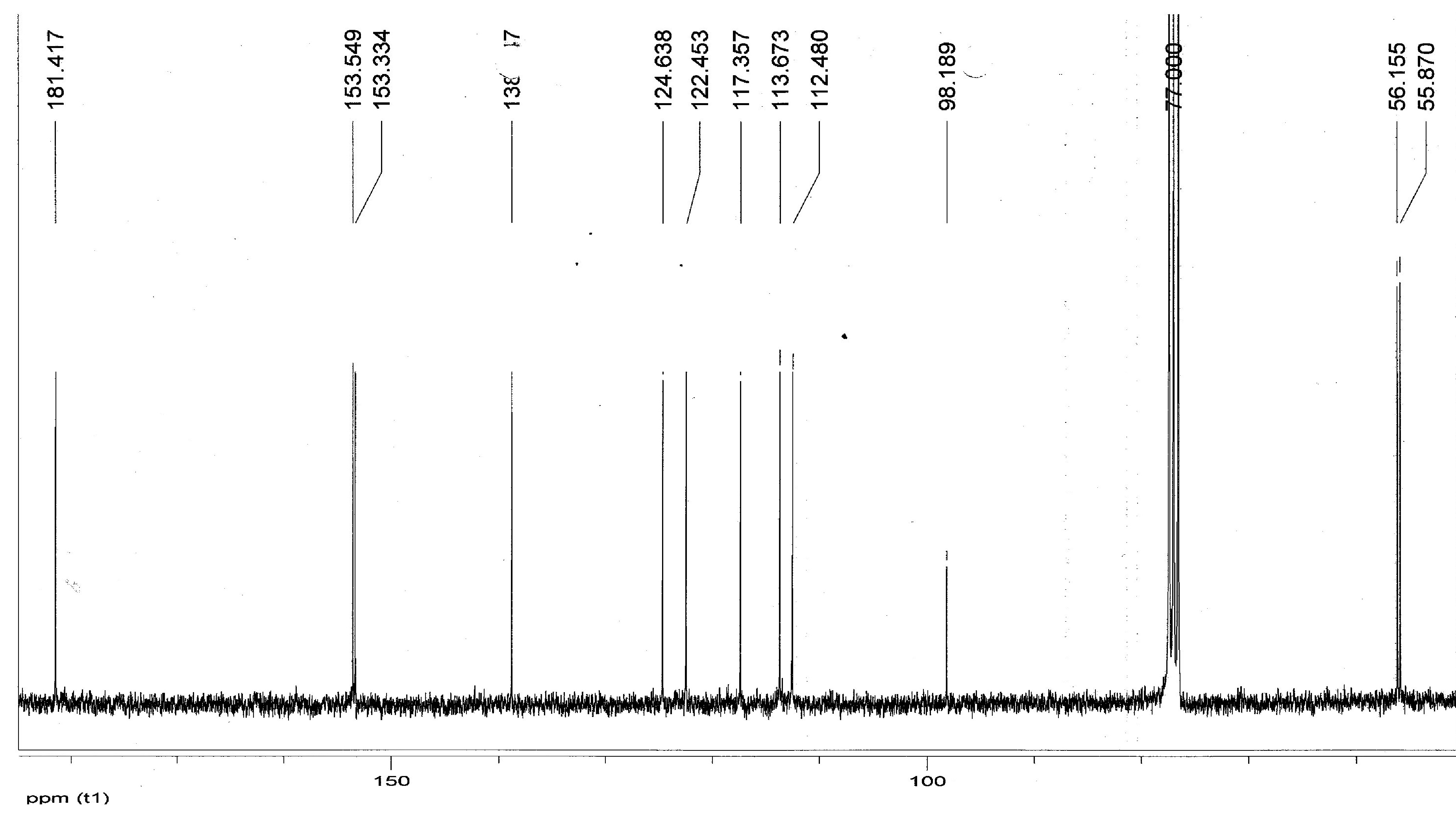
**

(1E,6E)-1,7-bis(2,6 -dimethoxyphenyl)-4-bromo-1,6-heptadiene-3,5-dione (**4d**)

(1E,6E)-1,7-bis(3,4-dimethoxyphenyl)-4-bromo-1,6-heptadiene-3,5-dione (**4e**)

(1E,6E)-1,7-bis(3,5-dimethoxyphenyl)-4-bromo-1,6-heptadiene-3,5-dione (**4f**)
